# Defect passivation and crystallization modulation in methylammonium-free wide-bandgap perovskites for all-perovskite tandem solar cells

**DOI:** 10.1126/sciadv.adv4501

**Published:** 2025-09-17

**Authors:** Xuefei Jia, Kaicheng Zhang, Xiaofeng Gao, Xufeng Liao, Yujie Yang, Weisheng Li, Xiaojing Lv, Xinyu Zhao, Jiali Liu, Yitong Ji, Zhongliang Yan, Qingguo Du, Fuzhi Huang, Zhiwei Ren, Yaxin Zhai, Wenchao Huang, Yang Bai, Canglang Yao, Qianqian Lin, Yi-Bing Cheng, Jinhui Tong

**Affiliations:** ^1^ State Key Laboratory of Advanced Technology for Materials Synthesis and Processing, Wuhan University of Technology, Wuhan 430070, P. R. China.; ^2^ Institute of Materials for Electronics and Energy Technology (i-MEET), Department of Materials Science and Engineering, Friedrich-Alexander-Universität Erlangen-Nürnberg, Martensstrasse 7, 91058 Erlangen, Germany.; ^3^ Key Lab of Artificial Micro- and Nano-Structures of Ministry of Education of China, School of Physics and Technology, Wuhan University, Wuhan 430072, P. R. China.; ^4^ Key Laboratory of Low-Dimensional Quantum Structures and Quantum Control of Ministry of Education, Department of Physics, Hunan Normal University, Changsha 410081, P. R. China.; ^5^ Faculty of Materials Science and Energy Engineering, Shenzhen University of Advanced Technology, Shenzhen 518107, China.; ^6^ School of information engineering, Wuhan University of Technology, Wuhan 430070, P. R. China.; ^7^ Foshan Xianhu Laboratory of the Advanced Energy Science and Technology, Guangdong Laboratory, Foshan 528216, P. R. China.; ^8^ Department of Electrical and Electronic Engineering, Photonics Research Institute (PRI), Research Institute for Intelligent Wearable Systems (iWEAR), The Hong Kong Polytechnic University, Hong Kong 100872, P. R. China.; ^9^ Laboratory of Advanced Materials, Fudan University, Shanghai 200438, P. R. China.

## Abstract

Wide-bandgap (WBG; >1.65 electron volts) perovskites based on iodine-bromine (I-Br) mixed halides are critical components of perovskite-based tandem solar cells (TSCs). However, the uncontrolled crystallization dynamics of Br-rich species lead to reduced grain sizes and high defect densities in WBG perovskite films. Herein, a multifunctional additive 3,4,5-trifluorobenzamide (TFBZ) was introduced to enhance the crystallinity and passivate defects of the methylammonium (MA)–free WBG perovskite films. The TFBZ demonstrates superior passivation capability compared to benzamide, effectively mitigating both iodine vacancies and undercoordinated Pb^2+^ defects via fluorine-enhanced interactions. In addition, the fluorine substituents in TFBZ could form N–H···F hydrogen bonds with formamidinium iodide to retard the crystallization rate of the perovskite. This proposed method is effective in defect passivation and crystal growth modulation for both 1.67– and 1.79–electron volt MA-free WBG perovskites, enabling the fabrication of MA-free all-perovskite TSCs with an encouraging power conversion efficiency of 29.01% (certified at 28.52%).

## Teaser

A well-designed multifunctional additive enabled the fabrication of high-performance methylammonium-free all-perovskite tandems.

## INTRODUCTION

Wide-bandgap (WBG; >1.65 eV) perovskites are essential components of various perovskite-based tandem solar cells (TSCs) (*1*–*3*). To optimize the performance of the whole tandem stacks, it is imperative to enhance the efficiency and stability of the WBG perovskite subcells. In recent years, methylammonium (MA)–free WBG perovskite films have attracted considerable attention due to their excellent thermal and phase stability. The removal of volatile MA components effectively suppresses ion migration and phase segregation in perovskites (*4*), improving device stability during prolonged operation. However, in MA-free perovskites, lattice distortion induced by the large-sized formamidinium cation (FA^+^; 253 pm) and the formation of uncontrolled intermediate phases disrupt crystallization kinetics, leading to structural defects such as pinholes and cracks (*5*–*7*). The high-bromine (Br) content in WBG perovskites promotes rapid crystallization during film formation (*8*). Due to Br’s low solubility (enhancing nucleation) and small ionic radius (accelerating grain coarsening) (*9*), the resulting films often exhibit reduced grain sizes, rough surfaces, and nonuniform halide phase distribution (*10*). The defect states at interfaces and grain boundaries serve as nonradiative recombination centers, causing severe charge carrier loss (*11*, *12*). Moreover, rough film surface commonly leads to poor contact between the perovskite layer and charge transport layer, increasing the interface resistance and hindering carrier extraction and transport (*13*, *14*). Critically, such structural defects also degrade long-term stability: Pinholes facilitate moisture and oxygen infiltration, defective grain boundaries accelerate ion migration, and inhomogeneous phase distribution triggers halide segregation (*15*). Thus, developing crystallization modulation and defect passivation strategies is essential for achieving efficient and stable MA-free WBG perovskites.

So far, various strategies aimed at reducing defect density and improving the crystallinity of perovskite films have been reported (*16*, *17*). Notably, additive engineering is perceived as a straightforward and efficacious approach to address the aforementioned challenges and enhance the performance of perovskite solar cells (PSCs). Among these additives, specific passivation groups such as amino (–NH_2_) and carbonyl (–C=O) are commonly used to interact with defect sites in perovskite films. For instance, Jiang *et al.* (*18*) enhanced the passivation capability for lead-based defects by modulating the spatial torsion angle of aromatic ketones, which increased the electron cloud density around carbonyl groups. In a separate study, 2-amino-4,5-imidazoledicarbonitrile was incorporated into WBG perovskite to suppress photoinduced iodine migration and phase segregation (*19*). Additionally, fluoroalkyl groups, valued for their strong electronegativity and hydrophobicity, have been incorporated into passivation materials to improve perovskite stability (*20*–*22*). However, current research primarily focuses on studying individual functional groups in isolation, while the synergistic mechanisms among multiple functional groups, particularly their combined effects on defect passivation and crystallization modulation, remain underexplored. This leaves great potential for tailoring multifunctional additives with robust passivation effects and crystallization modulation properties, enabling high-quality perovskite films with low defect densities and large grains to enhance the efficiency and stability of MA-free WBG PSCs.

In this work, we report a multifunctional additive: 3,4,5-trifluorobenzamide (TFBZ), which can modulate MA-free WBG perovskite crystallization and simultaneously passivate defects. First, the –NH_2_ and –C=O functional groups in TFBZ could coordinate with the perovskite to passivate defects. Second, the fluoride (F) substituents in TFBZ form a large molecular dipole, which enhances the passivation effects of –NH_2_ and –C=O groups. Third, the fluorine substituents form N–H···F hydrogen bonds with formamidinium iodide (FAI), slowing the crystallization of the WBG perovskite. The prolonged crystallization window facilitates the ordered growth of perovskite grains, reducing grain boundaries and pinholes while lowering defect density, thereby suppressing nonradiative recombination. Consequently, the optimized 1.67-eV MA-free WBG PSCs exhibit a champion power conversion efficiency (PCE) of 22.78% and an open-circuit voltage (*V*_OC_) of 1.28 V. The unencapsulated TFBZ-based device maintains 85% of its initial efficiency after 2400 hours under ambient air. This method was also effective for 1.79-eV MA-free WBG PSCs, achieving a PCE of 20.21% and a *V*_OC_ of 1.36 V. Meanwhile, MA-free all-perovskite TSCs with a champion PCE of 29.01% (certified at 28.52%) were obtained when integrated with 1.25-eV MA-free narrow-bandgap (NBG) perovskite subcells. This PCE is the highest reported value for MA-free all-perovskite TSCs. Furthermore, the TFBZ-based MA-free all-perovskite TSCs exhibited no more than 20% PCE loss after 800 hours of continuous illumination in N_2_.

## RESULTS

### Interactions between additives and WBG perovskite

**Fig. 1.**
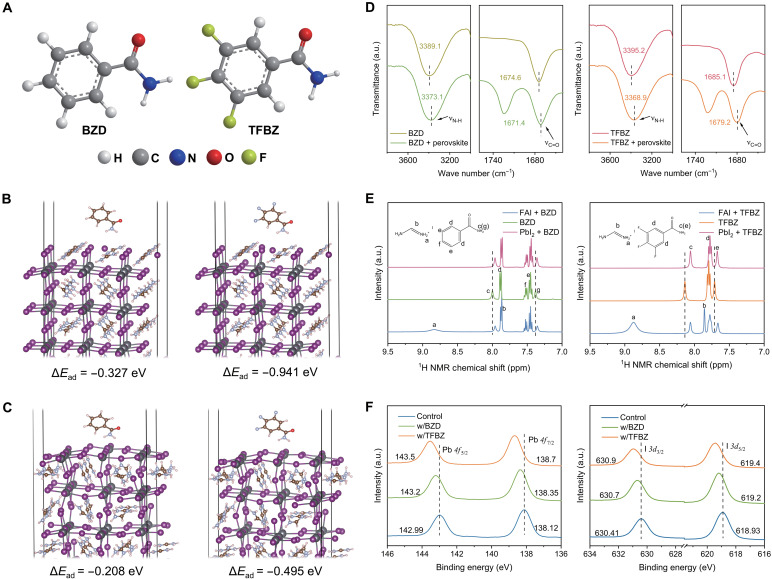
Molecular structures of additives and their interaction with perovskite. (**A**) Molecular structures of BZD and TFBZ. (**B**) Density functional theory (DFT) calculations of the adsorption energies between the FAI termination and BZD/TFBZ molecules. (**C**) DFT calculations of the adsorption energies between the PbI_2_ termination and BZD/TFBZ molecules. (**D**) Partial enlarged view of the Fourier transform infrared (FTIR) spectra for TFBZ and BZD, as well as their mixtures with perovskite. (**E**) Partial enlarged view of ^1^H nuclear magnetic resonance (NMR) spectra for BZD, TFBZ, and their mixtures with FAI and PbI_2_ in DMSO-d_6_ solvent. (**F**) X-ray photoelectron spectroscopy (XPS) spectra of Pb 4f and I 3d for perovskite films with and without TFBZ/BZD addition. a.u., arbitrary unit.

Designing anchoring groups that can facilitate strong absorption on perovskites is a promising approach for achieving controllable crystallization and excellent defect passivation (*23*). The benzamide (BZD) molecule comprises –NH_2_ and –C=O function groups, which can interact with the defect sites in perovskites to passivate traps (*24*). Here, we use TFBZ as an additive, which contains three F atoms at the para positions of –NH_2_ and –C=O (Fig. 1A). Theoretical calculations reveal that TFBZ has a larger dipole moment and greater molecular polarity than BZD (table S1), which suggests the possibility of stronger intermolecular interactions. Density functional theory (DFT) calculations were performed to compare the adsorption energies of BZD and TFBZ with the surface terminations of perovskite. The adsorption energy of TFBZ on the FAI termination (−0.941 eV) is three times stronger than that of BZD (−0.327 eV) (Fig. 1B). Similarly, TFBZ exhibits more favorable adsorption on PbI_2_ termination (−0.495 eV) compared to BZD (−0.208 eV) (Fig. 1C). To address potential compositional complexity, we further calculated the adsorption energies for FABr and PbBr_2_ terminations. Notably, both Br-containing terminations showed analogous trends, with TFBZ demonstrating consistently stronger adsorption on perovskite surfaces (fig. S1). These results demonstrate that fluorine substituents substantially enhance the interaction between TFBZ and perovskite components.

Fourier transform infrared (FTIR) spectroscopy was used to preliminarily investigate the interaction mechanism between TFBZ and perovskite. Figure 1D shows that the N–H stretching vibration peak of BZD shifts from 3389.1 to 3373.1 cm^−1^ after mixing with perovskite, and the C=O stretching vibration peak shifts from 1674.6 to 1671.4 cm^−1^. For TFBZ, the N–H stretching vibration peak shifts from 3395.2 to 3368.9 cm^−1^, and the C=O peak moves from 1685.1 to 1679.2 cm^−1^. The larger shifts of the N–H and C=O stretching vibration peaks of TFBZ indicate that TFBZ can coordinate with perovskite more robustly than BZD, which is consistent with the DFT calculations. The shifts of the C=O stretching vibration peaks are attributed to their interaction with uncoordinated Pb^2+^ (*25*). The shifts of the N–H stretching vibration peaks may result from the formation of N–H···I hydrogen bonds between –NH_2_ groups and perovskite components, which leads to electron delocalization in the N–H bonds (*26*). ^1^H nuclear magnetic resonance (NMR) spectroscopy further confirmed the hydrogen bonding between the additives and perovskite. As shown in Fig. 1E, the BZD exhibits characteristic peaks at 8.00 and 7.38 parts per million (ppm), corresponding to its –NH_2_ groups. Upon mixing BZD with FAI, these –NH_2_ peaks shift to 7.96 and 7.35 ppm, respectively, while mixing with PbI_2_ results in shifts to 7.96 and 7.36 ppm. Similarly, the –NH_2_ peaks of TFBZ appear at 8.14 and 7.71 ppm. When TFBZ is mixed with FAI, the corresponding –NH_2_ peaks shift to 8.05 and 7.66 ppm, whereas mixing with PbI_2_ induces shifts to 8.05 and 7.67 ppm. The observed chemical shift indicates the formation of N–H···I hydrogen bonds between the additives and perovskite components, which can effectively anchor iodide ions, thereby suppressing the formation of iodide vacancies (*27*). Notably, the more pronounced peak shifts observed for TFBZ mixtures demonstrate stronger hydrogen bonding with perovskite compared to BZD. The chemical states of primary elements on the perovskite surface upon interaction with additives were investigated by x-ray photoelectron spectroscopy (XPS) (Fig. 1F and fig. S2). The peaks associated with I 3d in the control perovskite occur at 630.41 eV (I 3d_3/2_) and 618.93 eV (I 3d_5/2_). After BZD addition, the I 3d_3/2_ and I 3d_5/2_ peaks shifted to 630.7 and 619.2 eV, respectively. Upon TFBZ incorporation, the peaks further shifted to 630.9 eV (I 3d_3/2_) and 619.4 eV (I 3d_5/2_). This shift can be attributed to hydrogen bond formation between the perovskite and the –NH_2_ group, as further evidenced by the corresponding displacement of the N–H bond peak in TFBZ after mixing with perovskite (fig. S3). Similar peak shifts were observed for Pb 4f upon BZD and TFBZ addition, confirming that TFBZ exhibits stronger interaction with uncoordinated Pb^2+^, consistent with FTIR and DFT results. Iodine vacancies and undercoordinated Pb^2+^ in perovskite films are the primary sources of nonradiative recombination (*28*). The enhanced interaction between TFBZ and perovskite can effectively suppress the formation of uncoordinated Pb^2+^ and iodide vacancies, thereby improving carrier lifetime.

We then analyzed the interaction between the fluorine groups in TFBZ and perovskite using ^19^F NMR. After TFBZ mixes with FAI, ^19^F shifts to a higher δ value (fig. S4), which is attributed to the N–H···F hydrogen bonds between the fluorine atoms of TFBZ and the FA^+^ cations. This interaction withdraws electron density from the fluorine atoms, reducing their shielding effect and resulting in the observed chemical shift (*29*). The N–H···F hydrogen bonds between TFBZ and FA^+^ can effectively suppress the formation of cation vacancies, thereby enhancing the stability of perovskite films.

### Modulation of WBG perovskite crystallization

**Fig. 2.**
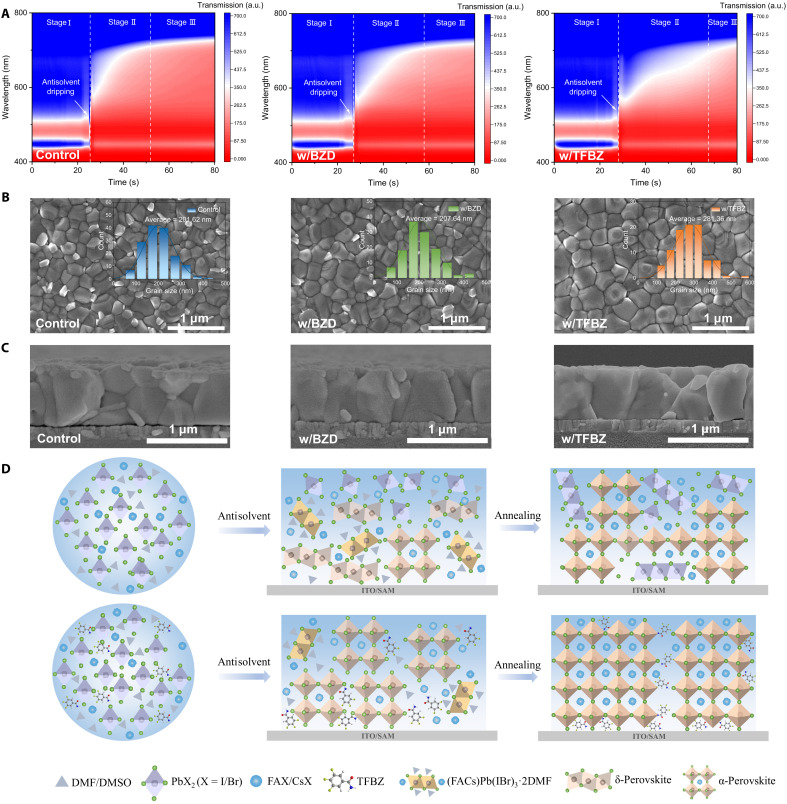
Effect of additives on the nucleation, crystallization, and morphology of perovskite films. (**A**) 2D contour maps of in situ UV-vis transmission spectra for the control, w/BZD, and w/TFBZ during spin coating. (**B**) Top-view scanning electron microscopy (SEM) images of control, w/BZD, and w/TFBZ perovskite films. The insets show the grain size distributions. (**C**) Cross-sectional SEM images of control, w/BZD, and w/TFBZ perovskite films. (**D**) Schematic illustration of the crystallization processes in control and TFBZ-modified perovskite films. a.u., arbitrary unit.

The interaction between additives and perovskite has the potential to affect the crystallization process of the perovskite (*30*). To evaluate the effect of BZD and TFBZ on the WBG perovskite crystallization, in situ ultraviolet-visible (UV-Vis) transmission and in situ photoluminescence (PL) measurements were conducted (fig. S5). The in situ UV-vis spectra reveal three distinct stages of perovskite film formation: (I) a liquid stage with negligible absorbance, (II) a nucleation and crystal growth stage with intensifying absorbance, (III) a solid-film stage with stabilized absorbance (*31*). Compared with the control and BZD-based (denoted as w/BZD) perovskite films, the TFBZ-based (denoted as w/TFBZ) perovskite film exhibited a prolonged nucleation and crystallization process after chlorobenzene (CB) antisolvent dripping (Fig. 2A). The crystallization time was quantified by monitoring the time-dependent PL intensity at 740 nm (photon energy of 1.67 eV). The PL intensity maxima for the control, w/BZD, and w/TFBZ films were observed at 28.98, 31.47, and 38.71 s after antisolvent dripping, respectively, corresponding to their respective crystallization completion times (fig. S6). This crystallization-modulating effect was also observed during the annealing process. Time-dependent UV-vis transmittance analysis at 600 nm revealed that the w/TFBZ film required 27.41 s to complete crystallization, which was longer than both the control film (19.54 s) and the w/BZD film (20.63 s) (fig. S7). This may be attributed to the strong hydrogen bonding between TFBZ and FA^+^, which inhibits the direct reaction of PbI_2_ and PbBr_2_ with organic ammonium salts, thus retarding the perovskite crystallization rate (*5*).

To further investigate the role of TFBZ in the perovskite film formation process, the wet films were characterized by x-ray diffraction (XRD) before annealing. As shown in fig. S8, the control wet film shows distinct peaks of nonphotoactive δ-phase and intermediate phases, which may result in nonuniform grain formation and a rough surface, thereby reducing the efficiency of PSCs (*32*). In comparison, the α-phase signal in the w/TFBZ wet film is stronger, with almost no δ-phase detected, indicating that the TFBZ additive can effectively reduce the residual δ-phase and enhance the phase purity of the final film. The XRD results of the annealed perovskite films reveal less residual crystalline PbI_2_ phase in the w/TFBZ film (fig. S9), indicating enhanced conversion efficiency of PbI_2_ during perovskite crystallization. Besides, w/TFBZ film exhibits a smaller full width at half maximum for the (100) peak (0.107°) compared to the control film (0.154°) and w/BZD film (0.136°), indicating the larger grain sizes of the w/TFBZ film (table S2). Figure S10 presents the two-dimensional (2D) grazing-incidence wide-angle x-ray scattering (GIWAXS) data and the corresponding 1D out-of-plane radial cake cut profiles of the perovskite films. The strongest isotropic ring located at ~10 nm^−1^ corresponds to the (100) crystallographic plane of the perovskite structure, while the faint out-of-plane diffraction arc in the low-*q* region around 9 nm^−1^ is associated with PbI_2_. Compared to the control and w/BZD films, the w/TFBZ film exhibits a stronger intensity of the (100) peak, indicating that the TFBZ additive enhances the crystallinity of the perovskite, thereby facilitating charge carrier transport. Moreover, the attenuated PbI_2_ characteristic scattering signal in the w/TFBZ film, consistent with XRD observations, suggests reduced carrier recombination at grain boundaries.

The morphology of the control, w/BZD, and w/TFBZ perovskite films was investigated using scanning electron microscopy (SEM) and atomic force microscopy (AFM). As shown in Fig. 2B, distinct white PbI_2_ phases were observed in the control and w/BZD films, likely due to incomplete precursor conversion during rapid crystallization. Residual PbI_2_ on the surface can act as nonradiative recombination centers and accelerate moisture-induced degradation, ultimately impairing device efficiency and stability (*33*). In contrast, the w/TFBZ perovskite film exhibited enlarged, uniformly distributed grains and the disappearance of the PbI_2_ phase. The formation of large-sized grains can be attributed to TFBZ-induced retardation of crystallization kinetics, which reduces instantaneous nucleation density and crystallization rate, thereby favoring lateral grain expansion and coalescence (*34*). Energy-dispersive x-ray spectroscopy analysis shows that TFBZ is primarily distributed at the grain boundaries (fig. S11). This distribution correlates with the suppression of PbI_2_ formation at these sites, likely resulting from coordination between TFBZ’s carbonyl group and undercoordinated Pb^2+^ ions, which inhibits PbI_2_ precipitation. Cross-sectional SEM images revealed that the control and w/BZD perovskite films consisted of granular crystals stacked together, whereas the w/TFBZ perovskite film displayed vertically aligned through-growth grains (Fig. 2C). This indicates that TFBZ effectively improves the quality of the perovskite film. The reduced grain boundaries and vertically grown grains facilitate the transport of photogenerated charge carriers. UV-vis spectroscopy analysis shows a slight increase in the absorption intensity of the w/TFBZ film, possibly due to more uniform crystal growth and improved film quality (fig. S12). AFM images reveal distinct variations in film roughness, with the root-mean-square roughness values of the control, w/BZD, and w/TFBZ films being 30.5, 26.6, and 22.2 nm, respectively (fig. S13). The reduced surface roughness indicates decreased nonradiative recombination sites at the interface. The smoother surface also facilitates better contact between the perovskite layer and electron transport layer, promoting charge extraction and efficiency collection. On the basis of the above results, we speculate that the incorporation of TFBZ additive can delay crystallization through hydrogen bonding interactions and suppress the formation of nonphotoactive δ-phase and residual PbI_2_, thereby improving the quality of the perovskite layer. The crystallization-modulating effect of the TFBZ is illustrated in Fig. 2D.

Time-of-flight secondary-ion mass spectrometry (TOF-SIMS) analysis reveals a gradual increase in F^−^ signal intensity from the top surface to the bottom interface in the perovskite film (fig. S14), as the large molecular size TFBZ was driven downward during the top-down crystallization process of the perovskite (*13*). The graded distribution of TFBZ enables optimal passivation at the buried interface where defects are most deleterious. To investigate the effect of additives on the buried interface, we peeled off the perovskite films from the substrates (fig. S15). SEM images reveal that the w/TFBZ film exhibits a smoother bottom surface, larger grain size, and less residual PbI_2_ (fig. S16), which can collectively lower interfacial resistance and improve charge collection.

### Reduction of trap density in WBG perovskite films

**Fig. 3.**
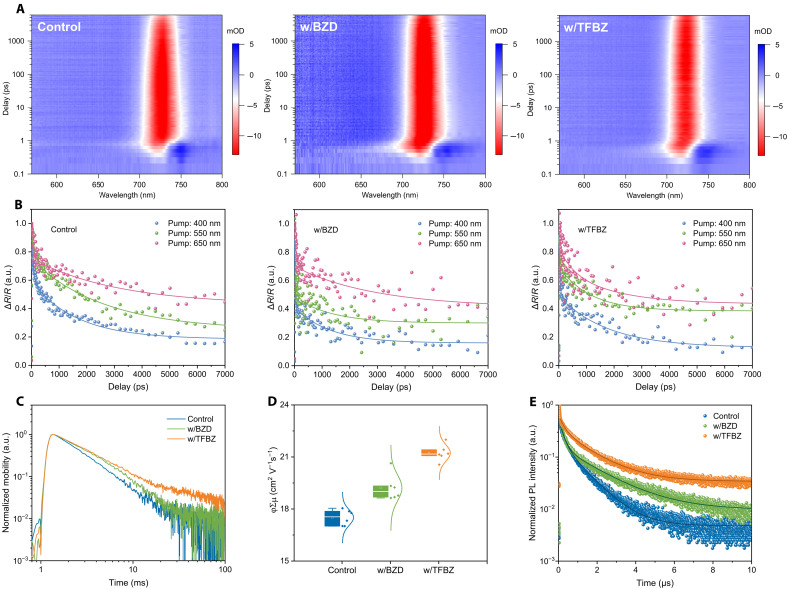
Carrier dynamics and nonradiative recombination of WBG perovskite thin films. (**A**) The pseudocolor representation of TA spectral evolution for the control, w/BZD, and w/TFBZ perovskite films. (**B**) Normalized surface carrier kinetics of the control, w/BZD, and w/TFBZ perovskite films photoexcited at the indicated wavelength (solid traces are nonlinear least-squares global best-fit curves). (**C**) Time-resolved microwave conductivity (TRMC) decays of the control, w/BZD, and w/TFBZ perovskite films. (**D**) Charge carrier mobilities of the control, w/BZD, and w/TFBZ perovskite films. (**E**) TRPL spectra of the control, w/BZD, and w/TFBZ perovskite films. a.u., arbitrary unit.

Transient absorption (TA) spectroscopy was used to investigate the excited-state carrier dynamics in control, BZD-based, and TFBZ-based perovskite films, with a temporal resolution spanning from 0.1 to 1000 ps. All films exhibited a photobleaching (PB) peak at 730 nm (Fig. 3A). Notably, the w/TFBZ film demonstrated excellent spectral stability, exhibiting negligible PB peak shift within the 1000-ps timeframe, indicating its uniform composition and homogeneous photogenerated carriers (*35*). Furthermore, both the control and w/BZD film show substantial PB signal decay, demonstrating fast recombination of photogenerated carriers at the PB position. The PB signal of the w/TFBZ film remains stable, indicating that FBZ effectively passivates defect states in the perovskite film, thereby suppressing nonradiative recombination and extending charge carrier lifetimes. Subsequently, the carrier diffusion and recombination process on the surfaces (~20 nm) of all three perovskite films is studied by performing TA spectroscopy in reflection geometry [i.e., transient reflection (TR) measurements]. The dynamics of surface carriers are predominantly influenced by two processes: surface recombination and diffusion into the bulk. This method facilitates the simultaneous quantification of two crucial parameters, the diffusion coefficient (*D*) and surface recombination velocity (*S*), by modeling the distinct surface carrier kinetics through a comprehensive fitting procedure. In this study, we used a 1D diffusion equation, incorporating the initial and boundary conditions specific to thin films

$$\frac{\partial N\left(x,t\right)}{\partial t}=D\frac{\partial^{2}N\left(x,\;t\right)}{\partial x^{2}}-\frac{N\left(x,t\right)}{\tau_{B}}$$ (1)

$${N\left(x,t\right)|}_{t=0}=N_{0}e^{-\mathrm{ax}}$$ (2)

$$\begin{array}{c} {\left.\frac{\partial N\left(x,t\right)}{\partial t}\right|}_{x=0}=\frac{S}{D}N\left(0,t\right)\;{\left.\frac{\partial N\left(x,t\right)}{\partial t}\right|}_{x=L_{\mathrm{PC}}}=-\frac{S}{D}N\left(L_{\mathrm{PC}},t\right) \end{array}$$ (3)

In the given equation, *N* represents the carrier concentration, *D* denotes the diffusion coefficient, τ_B_ signifies the bulk carrier lifetime, *N*_0_ is the initial surface carrier concentration, α is the absorption coefficient at the chosen photon energy, *L_PC_* indicates the thickness of the film, and *S* stands for the surface recombination velocity. The carrier recombination can be considered negligible on a 7-ns timescale; therefore, the second term in Eq. 1 is disregarded. By selecting varying pump photon energies, the absorption coefficient α in the initial condition equation (Eq. 2) is changed. A higher pump photon energy induces a larger gradient of the initial photocarrier density, and the TR dynamics decay faster. Three different pump wavelengths are used; the best-fit curves successfully reproduce the TR kinetics in all three perovskite films, as shown by the solid lines in Fig. 3B. The parameters are listed in table S3; the control film exhibits a *D* of 1.03 × 10^−5^ m^2^ s^−1^ and an *S* of 12.20 ms^−1^. After adding BZD, *D* increases to 4.36 × 10^−5^ m^2^ s^−1^, and *S* decreases to 7.51 ms^−1^, indicating improved charge transport and reduced surface recombination. *D* increases to 7.18 × 10^−5^ m^2^ s^−1^, and *S* decreases to 2.92 ms^−1^ for the w/TFBZ perovskite film, showing the highest diffusion efficiency and the lowest recombination rate among the three films.

The dynamics of photogenerated carrier migration and recombination in the perovskite films were characterized using time-resolved microwave conductivity (TRMC) (Fig. 3, C and D). The carrier diffusion lengths (*L*_D_) of perovskite films for the control, w/BZD, and w/TFBZ are 6.96, 7.12, and 7.70 μm, respectively, while their corresponding carrier mobilities are 17.51, 19.22, and 21.23 cm^2^ V^−1^ s^−1^ (table S4). Compared to the control film and w/BZD film, the w/TFBZ film exhibits prolonged carrier lifetime and enhanced carrier mobility, indicating that TFBZ effectively suppresses nonradiative recombination of photogenerated carriers in the perovskite film. Time-resolved PL (TRPL) and steady-state PL measurements were performed at both the top and bottom surfaces to further investigate the recombination dynamics. The TRPL results for the top surface reveal reduced nonradiative recombination in the TFBZ-based perovskite film, with the carrier lifetime of w/TFBZ film (1.58 μs) being much higher than that of w/BZD film (1.21 μs) and the control film (0.83 μs) (Fig. 3E and table S5). Furthermore, the TFBZ-based perovskite exhibited a substantially prolonged carrier lifetime at the bottom interface (fig. S17), confirming the effective suppression of trap states by TFBZ at the buried interface. The w/TFBZ film also demonstrated stronger PL emission intensity at both the top and bottom surfaces compared to the control and w/BZD films (fig. S18). All these results indicate lower trap density and inhibited nonradiative recombination in the w/TFBZ perovskite film (*36*). PL quantum yield (PLQY) measurements were performed to quantify the nonradiative recombination losses in the perovskite films. The w/TFBZ film exhibits a higher PLQY (2.021%) compared to the control film (0.661%) and w/BZD film (1.273%) (fig. S19), demonstrating the superior capability of TFBZ in suppressing defect-assisted nonradiative recombination. This enhancement is likely attributed to the improved crystallinity of the perovskite film (*37*). The trap densities of electron-only devices [indium tin oxide (ITO)/C_60_/perovskites/C_60_/silver (Ag)] and hole-only devices {ITO/[4-(3,6-dimethyl-9*H*-carbazol-9-yl)butyl]phosphonic acid (Me-4PACz)/perovskites/poly[bis(4-phenyl)(2,4,6-trimethylphenyl)amine] (PTAA)/Ag} were evaluated using space-charge limited current measurements (fig. S20). The trap density (*N*_t_) was determined from the trap-filled limit voltage (*V*_TFL_). Compared to the control film, both electron-only and hole-only devices based on BZD and TFBZ exhibit lower trap densities, with the TFBZ-based film showing the lowest trap density. The reduced trap density is attributed to the suppression of iodine and cation vacancies in the perovskite lattice.

### Stability of the WBG perovskite films

**Fig. 4.**
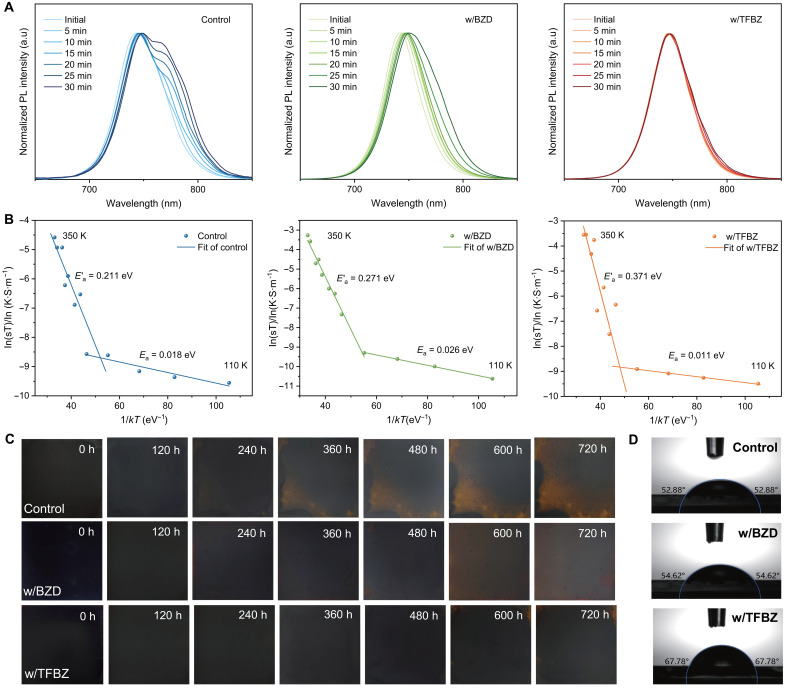
Stability of WBG perovskite thin films. (**A**) PL spectra of the control, w/BZD, and w/TFBZ perovskite films under continuous illumination for different times. (**B**) Ion migration activation energy of the control, w/BZD, and w/TFBZ perovskite films. (**C**) The evolution of photographs of the WBG perovskite films stored at 25°C and under 85% relative humidity (RH). (**D**) Water contact angles of the control, w/BZD, and w/TFBZ perovskite films. h, hours. a.u., arbitrary unit.

To evaluate the effectiveness of our strategy on the photostability of perovskite films, time-dependent PL measurements were performed under continuous illumination. The PL spectrum peaks of the control and w/BZD film exhibit a distinct red shift (Fig. 4A), indicating halide phase segregation. In contrast, the w/TFBZ film shows a negligible PL peak shift, indicating suppressed light-induced phase segregation. To further verify this, ion migration energy (*E*_a_) was studied using the temperature-dependent conductivity measurement. The ion migration energy is obtained based on the Nernst-Einstein relation: $\sigma \left(T\right)=\frac{\sigma_{0}}{T}\text{exp}\left(-\frac{E_{a}}{k_{B}T}\right)$ , where σ(T) is the conductivity, *T* denotes the temperature, σ_0_ is a constant, *k*_B_ is the Boltzmann constant, σ is ionic conductivity, and *E*_a_ is the ion migration activation energy (*38*). The *E*_a_ values calculated from the slope of the fitted line at relatively high temperatures for the control, w/BZD, and w/TFBZ films are 0.211, 0.271, and 0.371 eV, respectively (Fig. 4B). The higher *E*_a_ of the w/TFBZ film indicates suppressed halide migration, which is attributed to the reduction of halogen ion vacancies in the perovskite lattice, thereby decreasing the pathways for ion migration (*39*).

Additionally, the effect of the TFBZ additive on the humidity stability of WBG perovskite films was evaluated. Figure 4C shows the evolution of photographs of the WBG perovskite films at 25°C and under a relative humidity (RH) of 85%. The control film starts yellowing at 240 hours. In contrast, the black phases of the w/BZD and w/TFBZ perovskite films show prolonged durability. The w/BZD perovskite film turns yellow within 360 hours, while the w/TFBZ film maintains its black phase for more than 720 hours. The results demonstrate that the TFBZ additive enhances the humidity stability of perovskite films, owing to the hydrophobic nature of its fluorine substituents, as confirmed by water contact angle measurements (Fig. 4D).

### Photovoltaic performance of WBG PSCs and all-perovskite TSCs

**Fig. 5.**
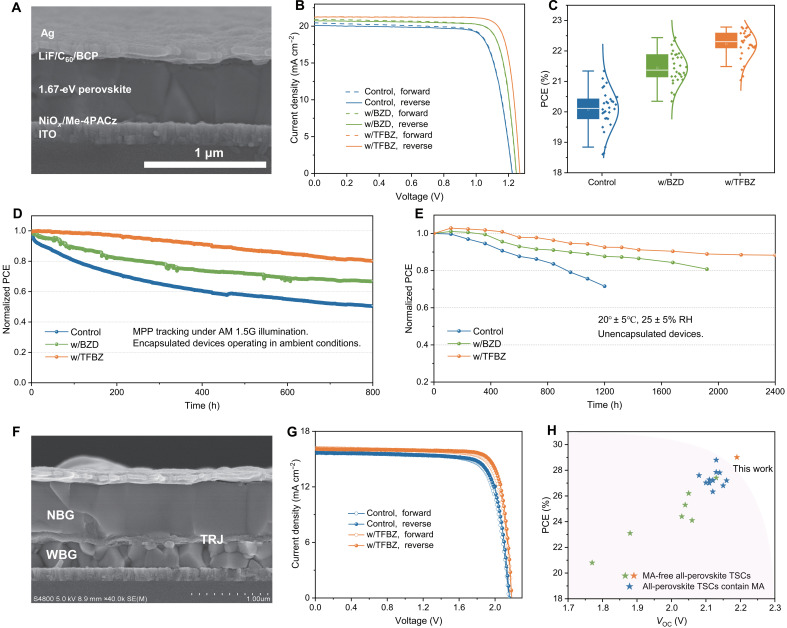
Structure and performance of MA-free WBG perovskite and all-perovskite tandem devices. (**A**) Cross-sectional SEM image of the 1.67-eV PSC device with the following structure: ITO/NiO*_x_*/Me-4PACz/perovskite/LiF/C_60_/BCP/Ag. (**B**) *J-V* curves of the best-performing devices for control, w/BZD, and w/TFBZ. (**C**) Statistical box plot of PCEs. (**D**) MPP test performed under a xenon lamp without filters at 1 sun for the encapsulated control, w/BZD, and w/TFBZ devices. (**E**) Ambient stability test performed under 25° ± 5°C and 20 ± 5% RH for the unencapsulated control, w/BZD, and w/TFBZ devices. (**F**) Cross-sectional SEM image of the MA-free all-perovskite tandem device. TRJ, tunnel recombination junction. (**G**) *J-V* curves of the best-performing all-perovskite tandem devices without and with TFBZ. (**H**) Comparison of PCE and *V*_OC_ of the champion device achieved in this work with reported high-performance all-perovskite tandem devices (source data are from tables S12 and S13).

We then fabricated 1.67-eV inverted MA-free WBG PSCs on the basis of the following device structure: ITO/nickel oxide (NiO*_x_*)/Me-4PACz/Cs_0.15_FA_0.85_Pb(I_0.77_Br_0.23_)_3_/lithium fluoride (LiF)/C_60_/bathocuproine (BCP)/Ag. Figure 5A shows the corresponding cross-sectional SEM image of the PSC. Compared with the control device, both the BZD and TFBZ additives improve the performance of PSCs (Fig. 5B). Champion device using TFBZ additive achieves a PCE of 22.78%, with a *V*_OC_ of 1.27 V, a fill factor (FF) of 84.52%, and a short-circuit current density (*J*_SC_) of 21.21 mA cm^−2^ (Table 1). In contrast, the efficiencies of the control and w/BZD WBG PSCs are 19.26 and 21.17%, with the *V*_OC_ of 1.22 and 1.25 V, FF of 78.39 and 81.74%, and *J*_SC_ of 20.09 and 20.74 mA cm^−2^, respectively. The optimal molar concentrations of BZD and TFBZ additives are 0.1 and 0.12%, respectively (fig. S21 and tables S6 and S7). Besides, the w/TFBZ PSC has a higher external quantum efficiency (EQE) value (fig. S22), with its integrated *J*_SC_ increasing from 20.02 to 20.80 mA cm^−2^. The photovoltaic (PV) parameter statistics of the control, w/BZD, and w/TFBZ devices further confirm the effectiveness of TFBZ in improving the performance of WBG PSCs (Fig. 5C and fig. S23).

**Table 1.** Photovoltaic parameters of the 1.67-eV WBG PSCs shown in Fig. 5B. The devices were measured with an active area of 0.09 cm^2^ under 1-sun illumination.

Device	Scan direction	*V*_OC_ (V)	*J*_SC_ (mA cm^−2^)	FF (%)	PCE (%)
Control	Reverse	1.22	20.09	78.39	19.26
	Forward	1.22	20.46	77.44	19.40
w/BZD	Reverse	1.25	20.74	81.74	21.17
	Forward	1.25	20.92	80.91	21.12
w/TFBZ	Reverse	1.27	21.21	84.52	22.78
	Forward	1.27	21.22	84.51	22.78

To gain more insights into the improved performance of the w/TFBZ PSCs, a series of characterizations was performed. To quantitatively analyze the impact of the TFBZ additive on the *V*_OC_ loss of devices, electroluminescence EQE (EQE_EL_) was characterized by operating PSCs as light-emitting diodes. The voltage loss Δ*V*_3_ caused by nonradiative recombination can be calculated using highly sensitive EQE (*40*). The EL intensity of the TFBZ-based device is twice that of the control, and Δ*V*_3_ decreases from 0.116 to 0.086 V (fig. S24 and table S8). These results demonstrate that TFBZ effectively suppresses nonradiative recombination in the perovskite layer, leading to a higher *V*_OC_ in the corresponding device.

To study the charge transport in PSCs, electrochemical impedance spectroscopy (EIS) was performed. The semicircle observed at low frequencies represents the complex resistance (*R*_rec_) at the interface between the perovskite and transport layer (*41*). Compared with the control device and w/BZD device, the w/TFBZ device has a higher *R*_rec_, indicating its superior carrier extraction capacity (fig. S25 and table S9). The built-in potential (*V*_bi_) of the devices was determined from Mott-Schottky (MS) measurements at 10 kHz. The w/TFBZ device exhibited a higher *V*_bi_ (1.08 V) compared to the control (1.0 V) and w/BZD (1.03 V) devices (fig. S25). This enhanced *V*_bi_ provides a stronger driving force for charge separation, consistent with the observed increase in *V*_OC_. The current density of the w/TFBZ device under dark conditions is more than one order of magnitude lower than that of the control device and w/BZD device (fig. S26). This indicates that a higher number of photogenerated carriers traverse through the device, rather than being directly shunted along defect-assisted channels. To further investigate charge carrier recombination, we analyzed the light intensity-dependent *V*_OC_ measurements to determine the ideality factor (*n*) of the PSCs (*10*). Compared to the control device and w/BZD device, the ideal factor of the w/TFBZ device is notably closer to 1 (fig. S27), indicating suppressed trap-assisted recombination (*42*). These results are consistent with the current density-voltage (*J-V*) characteristics, further confirming the improved charge transport and reduced defect density in the w/TFBZ film.

The characteristics of carrier transmission and extraction dynamics were further studied by measuring transient photovoltage (TPV) and transient photocurrent (TPC) (*43*). The TFBZ-based device exhibits slower voltage decay and faster current decay (fig. S28), indicating suppressed charge recombination and improved charge transport, thereby leading to improved *V*_OC_ and FF. UV photoelectron spectroscopy (UPS) reveals that the Fermi level of the control film, w/BZD film, and w/TFBZ film is -4.22, -4.08, and -4.21 eV, respectively (fig. S29). The upward shift of the Fermi level facilitates band bending, enhances charge carrier extraction, and further suppresses charge recombination at the interface (*44*). Thus, the reduction of traps at the interface and a better energy alignment may be the reasons behind the improved performance of w/TFBZ WBG PSCs.

When operating at the maximum power point (MPP), the stable power output (SPO) efficiencies reach 20.02, 21.15, and 21.61% for the control, BZD-based, and TFBZ-based devices, respectively, demonstrating the superior photostability of TFBZ-based devices (fig. S30). The operational stability of the encapsulated devices was further evaluated through MPP tracking under continuous simulated light illumination (100 mW cm^−2^). After 800 hours of illumination, the w/TFBZ device still maintained 80% of its initial efficiency (Fig. 5D), whereas the w/BZD and control devices dropped to 65 and 50%. The improved operational stability can be attributed to the synergistic effect of TFBZ in simultaneously enhancing crystallinity and passivating defects, which effectively suppresses light-induced phase segregation and inhibits interfacial nonradiative recombination in PSCs. Moreover, the w/TFBZ device exhibited better air stability than the control and w/BZD devices. After being stored in the air for 2400 hours, the w/TFBZ device still maintained over 85% of its initial efficiency (Fig. 5E). The improved air stability can be attributed to the enhanced hydrophobicity and reduced defect density of the w/TFBZ film, which effectively suppresses the phase segregation under moisture and oxygen exposure.

Encouraged by the improved efficiency and stability of the 1.67-eV MA-free WBG PSCs, 1.79-eV MA-free WBG PSCs were fabricated by using TFBZ as an additive, and the device structure is ITO/NiO_x_/Me-4PACz/Cs_0.2_FA_0.8_Pb(I_0.6_Br_0.4_)_3_/LiF/C_60_/BCP/Ag. As shown in fig. S31 and table S10, the best-performing TFBZ-based device achieved a champion PCE of 20.21%, surpassing the control device (18.07%). Compared to those PSCs with a bandgap of ~1.79 eV that have been reported, our device exhibits the highest PCE (table S11). The EQE spectrum (fig. S31) reveals an enhanced integrated current density, increasing from 16.96 to 17.45 mA cm^−2^. Furthermore, statistical analysis of the PCE distribution (fig. S31) confirms that the TFBZ additive effectively enhanced the photovoltaic performance of the 1.79-eV WBG PSCs.

By integrating the above 1.79-eV MA-free WBG perovskite with an MA-free Cs_0.25_FA_0.75_Pb_0.5_Sn_0.5_I_3_ NBG perovskite (~1.25 eV), we fabricated monolithic MA-free all-perovskite TSCs. The cross-sectional SEM image of the all-perovskite TSC is shown in Fig. 5F. As shown in Fig. 5G and Table 2, the PCE of the all-perovskite TSC was improved from 26.77 to 29.01% with the addition of TFBZ in the WBG perovskite. One such MA-free all-perovskite TSC was certified in a third-party organization and achieved a certified efficiency of 28.52% (fig. S32). This is the highest reported PCE value among MA-free all-perovskite TSCs (Fig. 5H and tables S12 and S13). The EQE is shown in fig. S33. The corresponding SPO is 25.90% for the control device and 28.93% for the TFBZ-based device (fig. S34), which closely aligns with the PCE obtained from *J-V* measurements. The evolution of PCEs of the unencapsulated all-perovskite tandem devices was measured under N_2_ conditions to determine the impact of TFBZ. After 800 hours of continuous illumination, the w/TFBZ device exhibited no more than 20% loss in PCE, while the PCE of the control device dropped to half of its initial value after 400 hours (fig. S34).

**Table 2.** Photovoltaic parameters of the all-perovskite TSCs shown in Fig. 5G. The devices were measured with an active area of 0.09 cm^2^ under 1-sun illumination.

Device	Scan direction	*V*_OC_ (V)	*J*_SC_ (mA cm^−2^)	FF (%)	PCE (%)
Control	Reverse	2.16	15.67	79.07	26.77
	Forward	2.15	15.87	76.73	26.23
w/TFBZ	Reverse	2.19	16.08	82.58	29.01
	Forward	2.19	16.20	79.58	28.18

## DISCUSSION

In summary, to address the critical challenges of poor crystallinity and high defect densities in MA-free WBG PSCs, we introduced a well-designed multifunctional additive, TFBZ. The fluorine atoms in TFBZ play an important role in enhancing the chemical bonding between the perovskite defect site and the formamide group in TFBZ, which exhibits a stronger passivation effect than BZD. Fluorine atoms also function as hydrogen bond acceptors and interact with FAI to form N–H···F hydrogen bonds, which retards the crystallization rate of the perovskites. The synergistic effect of fluorine atoms and the formamide group in TFBZ generates high-quality perovskite films with large grain sizes and low defect density. This method demonstrates broad applicability to MA-free WBG PSCs with bandgaps of both 1.67 and 1.79 eV. The resulting devices achieved remarkable PCEs of 22.78 and 20.21%, respectively, while simultaneously exhibiting enhanced operational stability. Even more impressively, by combining the efficient 1.79-eV MA-free WBG perovskite with a 1.25-eV MA-free NBG perovskite, we achieved an MA-free two-terminal all-perovskite tandem device with an encouraging PCE of 29.01%. Overall, these findings underscore the efficacy of our approach in strategically designing the functional groups in additives to enhance the chemical interactions. This approach simultaneously enhances crystallinity and reduces defect densities in MA-free WBG perovskite films, ultimately enabling high-performance single-junction MA-free WBG PSCs and all-perovskite TSCs with improved efficiency and long-term operational stability.

## MATERIALS AND METHODS

### Materials

Anhydrous solvents including *N*,*N*-dimethylformamide (DMF; 99.8%), dimethyl sulfoxide (DMSO; 99.9%), CB (99.8%), ethanol (99.5%), isopropyl alcohol (IPA; 99.5%) were all purchased from Sigma-Aldrich. Lead thiocyanate [Pb(SCN)_2_; 99.5%] was purchased from Sigma-Aldrich. FAI (99.99%) was purchased from Greatcell Solar Materials. Lead iodide (PbI_2_; 99.99%), tin iodide(II) (SnI_2_, 97%), tin fluoride(II) (SnF_2_; 98%), and Me-4PACz (99.0%) were purchased from Tokyo Chemical Industry Co. Ltd. cesium iodide (CsI; 99.999%), formamidinium Br (FABr; 99.9%), lead bromide (PbBr_2_; 99.99%), octylammonium bromide (OABr; 99.5%), and phenethylammonium bromide (PEABr; 99.5%) were purchased from Xian Yuri Solar Co. Ltd. NiO*_x_* was purchased from Advanced Election Technology Co. Ltd. Poly(3,4-ethylenedioxythiophene):polystyrene sulfonate (PEDOT:PSS; CLEVIOS PVP AI 4083) was purchased from Heraeus LLC. TFBZ was purchased from MedBio. BZD was purchased from Aladdin. For evaporating materials, LiF (99.99%) was purchased from Sigma-Aldrich. C_60_ and BCP were purchased from Xi’an Yuri Solar Co. Ltd. All chemicals were used directly as received.

### Preparation of perovskite precursor solutions

Perovskite precursor solution at 1.5 M was prepared by mixing 58.46 mg of CsI, 159.96 mg of FAI, 43.13 mg of FABr, 126.62 mg of PbBr_2_, 532.47 mg of PbI_2_ in 1 ml of mixture solvent (DMSO:DMF = 1:4 v/v) with a chemical formula of Cs_0.15_FA_0.85_Pb(I_0.77_Br_0.23_)_3_ to form a 1.67-eV perovskite. Perovskite precursor solution at 1.2 M was prepared by mixing 62.35 mg of CsI, 60 mg of FABr, 82.55 mg of FAI, 176.16 mg of PbBr_2_, 331.92 mg of PbI_2_, and 4.85 mg of Pb(SCN)_2_ in 1 ml of mixture solvent (DMSO:DMF = 1:3 v/v) with a chemical formula of Cs_0.2_FA_0.8_Pb(I_0.6_Br_0.4_)_3_ to form a 1.79-eV perovskite. To minimize defects in perovskite film, 0.12 mol % of TFBZ or 0.1 mol % of BZD was added to the two WBG perovskite precursor solutions. As for NBG perovskite films, 2.0 M Cs_0.25_FA_0.75_Pb_0.5_Sn_0.5_I_3_ precursor solution was prepared by dissolving 129.90 mg of CsI, 257.96 mg of FAI, 461.01 mg of PbI_2_, 372.52 mg of SnI_2_, and 15.67 mg of SnF_2_ in 1 ml of mixture solvent (DMSO:DMF = 1:4 v/v). The precursor solution was then stirred at room temperature for 2 hours. All precursors were filtered with 0.22-μm PTFE filters before use.

### Device fabrication of 1.67-eV WBG PSCs

The prepatterned ITO substrates were cleaned in an ultrasonic cleaner with acetone and IPA for 15 min, respectively. The ITO substrates were treated with UV ozone for 15 min. The NiO*_x_* nanoparticles (20 mg ml^−1^) dispersed in deionized water were spin coated onto the ITO substrate at 4000 rpm for 30 s and then annealed at 120°C for 10 min in ambient air. After that, Me-4PACz (0.5 mg ml^−1^) was spin coated on the ITO/NiO*_x_* at 3000 rpm for 30 s and then annealed at 100°C for 10 min in an N_2_-filled glove box. The preprepared Cs_0.15_FA_0.85_Pb(I_0.77_Br_0.23_)_3_ solution was spin coated on the substrates at 1000 rpm for 10 s and then at 5000 rpm for 30 s. CB (200 μl) was dripped onto the center of the wet film 15 s before the end of the spin-coating process. The perovskite films were immediately annealed at 100°C for 10 min on a hot plate. After cooling down to room temperature, 30 μl of OABr solution with a concentration of 1 mg ml^−1^ dissolved in IPA was dynamically spin coated on the perovskite film at 5000 rpm for 30 s and then annealed at 100°C for 10 min. All the spin-coating processes were conducted in an N_2_-filled glove box with a controlled temperature of 18° to 24°C, and the water and oxygen levels should be both controlled at less than 0.01 ppm. Last, 1-nm LiF at a rate of 0.02 Å s^−1^, 25-nm C_60_ at a rate of 0.5 Å s^−1^, 7-nm BCP at a rate of 0.5 Å s^−1^, and 100-nm Ag electrode at a rate of 1.0 Å s^−1^ were thermally evaporated, respectively, under high vacuum (<4 × 10^−6^ torr).

### Device fabrication of 1.79-eV WBG PSCs

The prepatterned ITO substrates were cleaned in an ultrasonic cleaner with acetone and IPA for 15 min, respectively. The ITO substrates were treated with UV ozone for 15 min. The NiO*_x_* nanoparticles (20 mg ml^−1^) dispersed in deionized water were spin coated onto the ITO substrate at 4000 rpm for 30 s and then annealed at 120°C for 10 min in ambient air. After that, Me-4PACz (0.5 mg ml^−1^) was spin coated on the ITO/NiO*_x_* at 3000 rpm for 30 s and then annealed at 100°C for 10 min in an N_2_-filled glove box. The preprepared Cs_0.2_FA_0.8_Pb(I_0.6_Br_0.4_)_3_ solution was spin coated at 500 rpm for 2 s and 4000 rpm for 60 s. CB was dripped onto the center of the wet film at 35 s before the end of spin coating. After that, the perovskite films were annealed at 65°C for 2 min, and 100°C for 10 min. After cooling down to room temperature, 30 μl of PEABr (2 mg ml^−1^ in IPA) was applied to the perovskite film and annealed at 100°C for 10 min. All the spin-coating processes were conducted in an N_2_-filled glove box with a controlled temperature of 18° to 24°C, and the water and oxygen levels should be both controlled at less than 0.01 ppm. The evaporation process is the same as the fabrication of the 1.67-eV device mentioned above.

### Device fabrication of monolithic all-perovskite TSCs

For the WBG front subcell, the NiO*_x_*, Me-4PACz, 1.79-eV bandgap perovskite, PEABr, LiF, and C_60_ were sequentially deposited in the same procedure as the WBG PSCs. Then, the SnO_2_ (30 nm)/Au (1 nm) tunnel recombination junction was deposited sequentially by the atomic layer deposition and thermal evaporation system onto the WBG subcell. After that, PEDOT:PSS was spin coated on top of the WBG subcell with 4000 rpm for 30 s. Then, the substrates were transferred onto a hot plate and annealed at 100°C for 20 min in air. After the cooling, the substrate was transferred to an N_2_-filled glove box to deposit the NBG perovskite subcell. The NBG perovskite precursor solution was spin coated on the WBG subcell at 5000 rpm for 80 s, during which 250 μl of CB was dripped onto the center of the wet film at 55 s before the end of spin coating. After that, the perovskite films were annealed at 120°C for 15 min. Then, 25-nm C_60_ at a rate of 0.5 Å s^−1^, 7-nm BCP at a rate of 0.5 Å s^−1^, and 100-nm Ag electrode at a rate of 1.0 Å s^−1^ were thermally evaporated, respectively, under high vacuum (<4 × 10^−6^ torr).

### Solar cell characterization and stability tests

*J-V* measurements were carried out using an Enli-Tech SS-X180R-3A solar simulator with air mass 1.5G spectrum and light intensity of 100 mW cm^−2^ in the nitrogen glove box at ~25°C. The PSCs were measured both in reverse and forward scan with a 10-mV interval and 10-ms delay time. The steady-state power output curves were recorded by tracing the current density at a bias (voltage at MPP). The device area was defined and characterized as 0.119 cm^2^ by a metal shadow mask. During *J-V* measurement, an optical aperture mask (9 mm^2^) was used to determine the cell area. The dark current density of devices was measured by a Keithley 2450 source meter. EQE measurements for PSCs were conducted by Enli-Tech QE-R measuring instrument. For EQE measurement of tandem cells, the WBG perovskite front subcells were measured with continuous light from a halogen lamp with an 800-nm polarized optical filter, while the NBG perovskite bottom subcells were measured with continuous light from a halogen lamp with a 500-nm polarized optical filter. For the MPP tracking test under illumination, we encapsulated the cell area with AB glue and epoxy resin. We fixed the device at the MPP voltage (*V*_mpp_) and recorded the change in current density at ~25°C and ~50% RH. The humidity stability and light stability of the devices were tested by the structure of Glass/ITO/NiO*_x_*/Me-4PACz/Perovskite/LiF/C_60_/SnO_2_/Ag. The long-term MPP tracking was carried out on a multichannel solar cells stability test system from Wuhan 91PVKSolar Technology Co. Ltd., China.

### TA and TR measurements

TA and TR measurements are conducted using a pump-probe spectrometer setup in transmission and reflection mode, respectively. Initially, a Ti:sapphire amplifier generates a fundamental laser pulse at 800 nm, operating at a repetition rate of 1 kHz. This fundamental pulse is then split into two branches by a beam splitter. One branch is directed toward an optical parametric amplifier to generate the pump pulse spanning from 290 to 2600 nm. The pump pulse, modulated at a frequency of 500 Hz, undergoes attenuation via neutral-density filter wheels. Simultaneously, the other branch of the fundamental pulse is focused into a sapphire crystal to produce a white-light continuum spanning from 350 to 1600 nm, used as the probe. Time delays between the pump and probe pulses (up to 8 ns) are achieved using a motorized translation stage with a retro-reflecting mirror. The pump and probe beams are spatially overlapped on the sample surface. In TA configuration both beams are incident on the sample normally. In TR configuration, the probe beam incident angle is fixed at 45°, while the pump beam is incident on the sample normally. The focused spot size at the sample position is ~200 μm for the probe beam and 600 μm for the pump beam.

### In situ UV-vis and PL measurements

The in situ UV-vis tests were carried out using a QE65 Pro UV-vis spectrometer (Ocean Optics). The sample was spin coated onto a glass substrate evaporated with 100 nm of Ag. During testing, the light from the tungsten light source passes through the optical fiber and convex lens before illuminating the sample and then is reflected by the Ag on the back of the glass onto the convex lens.

### Other characterizations

The FTIR spectroscopy was measured by Nicolet 6700, with a wavelength range of 4000 to 400 cm^−1^. The XPS and the UPS spectra were tested by ESCALAB 250Xi, Thermo Fisher Scientific K-Alpha. The SEM images were acquired using S-4800, HITACHI. The XRD patterns were obtained using a Bruker ECO D8 series. GIWAXS tests were performed by the GANESHA 300 XL SAXS system with an x-ray photon energy of 8.05 keV. AFM images were obtained using Bruker Dimension Icon XR equipment. PL and TRPL spectra were conducted by Micro Time 200 PicoQuant GmbH. For TRMC measurement, a FieldFox Handheld Microwave Analyzer (Keysight, N9915A) was applied as a microwave source and detector. UV-Vis absorption spectra were measured by lambda 750S, PerkinElmer. The EIS and MS plots were performed by using the electrochemical workstation EC-lab (SP300) in the dark. PLQY was tested by Enli-Tech with a 405-nm laser light source. The TOF-SIMS measurements were conducted with a PHI nano TOF III. A Bi^3++^ beam (30 kV, 2 nA, 200 μm) with a raster size of 100 μm was used as the primary beam to detect the samples, sputtering with an Ar^+^ beam (2 kV, 100 nA, 400 μm by 400 μm).

## Supplementary Materials

### This PDF file includes:

Figs. S1 to S34 Tables S1 to S13 References

## References

[1] Z. Zhang, W. Chen, X. Jiang, J. Cao, H. Yang, H. Chen, F. Yang, Y. Shen, H. Yang, Q. Cheng, X. Chen, X. Tang, S. Kang, X.-m. Ou, C. J. Brabec, Y. Li, Y. Li, Suppression of phase segregation in wide-bandgap perovskites with thiocyanate ions for perovskite/organic tandems with 25.06% efficiency. Nat. Energy 9, 592–601 (2024).

[2] T. Li, J. Xu, R. Lin, S. Teale, H. Li, Z. Liu, C. Duan, Q. Zhao, K. Xiao, P. Wu, B. Chen, S. Jiang, S. Xiong, H. Luo, S. Wan, L. Li, Q. Bao, Y. Tian, X. Gao, J. Xie, E. H. Sargent, H. Tan, Inorganic wide-bandgap perovskite subcells with dipole bridge for all-perovskite tandems. Nat. Energy 8, 610–620 (2023).

[3] D. Kim, H. J. Jung, I. J. Park, B. W. Larson, S. P. Dunfield, C. Xiao, J. Kim, J. Tong, P. Boonmongkolras, S. G. Ji, F. Zhang, S. R. Pae, M. Kim, S. B. Kang, V. Dravid, J. J. Berry, J. Y. Kim, K. Zhu, D. H. Kim, B. Shin, Efficient, stable silicon tandem cells enabled by anion-engineered wide-bandgap perovskites. Science 368, 155–160 (2020). 32217753 10.1126/science.aba3433

[4] A. J. Ramadan, R. D. J. Oliver, M. B. Johnston, H. J. Snaith, Methylammonium-free wide-bandgap metal halide perovskites for tandem photovoltaics. Nat. Rev. Mater. 8, 822–838 (2023).

[5] Y. Zheng, C. Tian, X. Wu, A. Sun, R. Zhuang, C. Tang, Y. Liu, Z. Li, B. Ouyang, J. Du, Z. Li, X. Wu, J. Chen, J. Cai, C.-C. Chen, Dual-interface modification for inverted methylammonium-free perovskite solar cells of 25.35% efficiency with balanced crystallization. Adv. Energy Mater. 14, 2304486 (2024).

[6] S. Wang, L. Tan, J. Zhou, M. Li, X. Zhao, H. Li, W. Tress, L. Ding, M. Graetzel, C. Yi, Over 24% efficient MA-free Cs_x_FA_1−x_PbX_3_ perovskite solar cells. Joule 6, 1344–1356 (2022).

[7] K. Wang, Z. Xu, Z. Guo, H. Wang, S. M. H. Qaid, K. Yang, Z. Zang, Phosphonate diacid molecule induced crystallization manipulation and defect passivation for high-performance inverted MA-free perovskite solar cells. Adv. Energy Mater. 14, 2402249 (2024).

[8] Y. An, N. Zhang, Z. Zeng, Y. Cai, W. Jiang, F. Qi, L. Ke, F. R. Lin, S.-W. Tsang, T. Shi, A. K. Y. Jen, H.-L. Yip, Optimizing crystallization in wide-bandgap mixed halide perovskites for high-efficiency solar cells. Adv. Mater. 36, e2306568 (2024). 37677058 10.1002/adma.202306568

[9] Q. Jiang, J. Tong, R. A. Scheidt, X. Wang, A. E. Louks, Y. Xian, R. Tirawat, A. F. Palmstrom, M. P. Hautzinger, S. P. Harvey, S. Johnston, L. T. Schelhas, B. W. Larson, E. L. Warren, M. C. Beard, J. J. Berry, Y. Yan, K. Zhu, Compositional texture engineering for highly stable wide-bandgap perovskite solar cells. Science 378, 1295–1300 (2022). 36548423 10.1126/science.adf0194

[10] R. Wang, X. Liu, S. Yan, N. Meng, X. Zhao, Y. Chen, H. Li, S. M. H. Qaid, S. Yang, M. Yuan, T. He, Efficient wide-bandgap perovskite photovoltaics with homogeneous halogen-phase distribution. Nat. Commun. 15, 8899 (2024). 39406749 10.1038/s41467-024-53344-9 PMC11480447

[11] F. Li, X. Deng, Z. Shi, S. Wu, Z. Zeng, D. Wang, Y. Li, F. Qi, Z. Zhang, Z. Yang, S.-H. Jang, F. R. Lin, S. W. Tsang, X.-K. Chen, A. K. Y. Jen, Hydrogen-bond-bridged intermediate for perovskite solar cells with enhanced efficiency and stability. Nat. Photonics 17, 478–484 (2023).

[12] J. Tao, C. Zhao, Z. Wang, Y. Chen, L. Zang, G. Yang, Y. Bai, J. Chu, Suppressing non-radiative recombination for efficient and stable perovskite solar cells. Energ. Environ. Sci. 18, 509–544 (2025).

[13] H. Gao, K. Xiao, R. Lin, S. Zhao, W. Wang, S. Dayneko, C. Duan, C. Ji, H. Sun, A. D. Bui, C. Liu, J. Wen, W. Kong, H. Luo, X. Zheng, Z. Liu, H. Nguyen, J. Xie, L. Li, M. I. Saidaminov, H. Tan, Homogeneous crystallization and buried interface passivation for perovskite tandem solar modules. Science 383, 855–859 (2024). 38386724 10.1126/science.adj6088

[14] S. Nagane, S. Macpherson, M. A. Hope, D. J. Kubicki, W. Li, S. D. Verma, J. Ferrer Orri, Y.-H. Chiang, J. L. MacManus-Driscoll, C. P. Grey, S. D. Stranks, Tetrafluoroborate-induced reduction in defect density in hybrid perovskites through halide management. Adv. Mater. 33, e2102462 (2021). 34219285 10.1002/adma.202102462 PMC11468984

[15] Y. Yao, B. Li, D. Ding, C. Kan, P. Hang, D. Zhang, Z. Hu, Z. Ni, X. Yu, D. Yang, Oriented wide-bandgap perovskites for monolithic silicon-based tandems with over 1000 hours operational stability. Nat. Commun. 16, 40 (2025). 39747820 10.1038/s41467-024-55377-6 PMC11696550

[16] Y. Yu, C. Wang, C. R. Grice, N. Shrestha, D. Zhao, W. Liao, L. Guan, R. A. Awni, W. Meng, A. J. Cimaroli, K. Zhu, R. J. Ellingson, Y. Yan, Synergistic effects of lead thiocyanate additive and solvent annealing on the performance of wide-bandgap perovskite solar cells. ACS Energy Lett. 2, 1177–1182 (2017).

[17] P. Pandey, S. Cho, J. Bahadur, S. Yoon, C.-M. Oh, I.-W. Hwang, H. Song, H. Choi, S. Hayase, J. S. Cho, D.-W. Kang, 4-Phenylthiosemicarbazide molecular additive engineering for wide-bandgap Sn halide perovskite solar cells with a record efficiency over 12.2%. Adv. Energy Mater. 14, 2401188 (2024).

[18] X. Jiang, L. Zhu, B. Zhang, L. Zheng, L. Wang, P. Li, M. Wang, G. Yang, K. Dong, S. Li, S. Liu, Y. Yin, H. Wang, S. M. Zakeeruddin, S. Pang, L. Sun, M. Grätzel, X. Guo, Spatial conformation engineering of aromatic ketones for highly efficient and stable perovskite solar cells. J. Am. Chem. Soc. 146, 34833–34841 (2024). 39648820 10.1021/jacs.4c13866

[19] X. Guo, Z. Jia, S. Liu, R. Guo, F. Jiang, Y. Shi, Z. Dong, R. Luo, Y.-D. Wang, Z. Shi, J. Li, J. Chen, L. K. Lee, P. Müller-Buschbaum, D. S. Ginger, D. J. Paterson, Y. Hou, Stabilizing efficient wide-bandgap perovskite in perovskite-organic tandem solar cells. Joule 8, 2554–2569 (2024).

[20] R. Sun, Q. Tian, M. Li, H. Wang, J. Chang, W. Xu, Z. Li, Y. Pan, F. Wang, T. Qin, Over 24% efficient poly(vinylidene fluoride) (PVDF)-coordinated perovskite solar cells with a photovoltage up to 1.22 V. Adv. Funct. Mater. 33, 2210071 (2023).

[21] Y. Yu, R. Liu, C. Liu, X.-L. Shi, H. Yu, Z.-G. Chen, Synergetic regulation of oriented crystallization and interfacial passivation enables 19.1% efficient wide-bandgap perovskite solar cells. Adv. Energy Mater. 12, 2201509 (2022).

[22] Y. Wang, R. Lin, C. Liu, X. Wang, C. Chosy, Y. Haruta, A. D. Bui, M. Li, H. Sun, X. Zheng, H. Luo, P. Wu, H. Gao, W. Sun, Y. Nie, H. Zhu, K. Zhou, H. T. Nguyen, X. Luo, L. Li, C. Xiao, M. I. Saidaminov, S. D. Stranks, L. Zhang, H. Tan, Homogenized contact in all-perovskite tandems using tailored 2D perovskite. Nature 635, 867–873 (2024). 39401514 10.1038/s41586-024-08158-6

[23] P. Hu, W. Zhou, J. Chen, X. Xie, J. Zhu, Y. Zheng, Y. Li, J. Li, M. Wei, Multidentate anchoring strategy for synergistically modulating crystallization and stability towards efficient perovskite solar cells. Chem. Eng. J. 480, 148249 (2024).

[24] S. Wang, P. Wang, B. Shi, C. Sun, H. Sun, S. Qi, Q. Huang, S. Xu, Y. Zhao, X. Zhang, Inorganic perovskite surface reconfiguration for stable inverted solar cells with 20.38% efficiency and its application in tandem devices. Adv. Mater. 35, e2300581 (2023). 37052233 10.1002/adma.202300581

[25] R. Wang, J. Xue, K.-L. Wang, Z.-K. Wang, Y. Luo, D. Fenning, G. Xu, S. Nuryyeva, T. Huang, Y. Zhao, J. L. Yang, J. Zhu, M. Wang, S. Tan, I. Yavuz, K. N. Houk, Y. Yang, Constructive molecular configurations for surface-defect passivation of perovskite photovoltaics. Science 366, 1509–1513 (2019). 31857483 10.1126/science.aay9698

[26] Z. Li, J. Wang, S. Lu, J. Liu, J. Zeng, H. Gao, C. Liu, W. Guo, Targeted synergistic chemical bonding strategy for efficient and stable CsPbI_3_-based perovskite solar cells. Chem. Eng. J. 499, 156691 (2024).

[27] S. Zhou, S. Fu, C. Wang, W. Meng, J. Zhou, Y. Zou, Q. Lin, L. Huang, W. Zhang, G. Zeng, D. Pu, H. Guan, C. Wang, K. Dong, H. Cui, S. Wang, T. Wang, G. Fang, W. Ke, Aspartate all-in-one doping strategy enables efficient all-perovskite tandems. Nature 624, 69–73 (2023). 37938775 10.1038/s41586-023-06707-z

[28] L. Lin, T. W. Jones, T. C.-J. Yang, X. Li, C. Wu, Z. Xiao, H. Li, J. Li, J. Qian, L. Lin, J. Q. Shi, S. D. Stranks, G. J. Wilson, X. Wang, Hydrogen bonding in perovskite solar cells. Matter 7, 38–58 (2024).

[29] Y. Kong, W. Shen, H. Cai, W. Dong, C. Bai, J. Zhao, F. Huang, Y. B. Cheng, J. Zhong, Multifunctional organic potassium salt additives as the efficient defect passivator for high-efficiency and stable perovskite solar cells. Adv. Funct. Mater. 33, 2300932 (2023).

[30] M. Li, R. Sun, J. Chang, J. Dong, Q. Tian, H. Wang, Z. Li, P. Yang, H. Shi, C. Yang, Z. Wu, R. Li, Y. Yang, A. Wang, S. Zhang, F. Wang, W. Huang, T. Qin, Orientated crystallization of FA-based perovskite via hydrogen-bonded polymer network for efficient and stable solar cells. Nat. Commun. 14, 573 (2023). 36732540 10.1038/s41467-023-36224-6 PMC9895431

[31] W. Yue, H. Yang, H. Cai, Y. Xiong, T. Zhou, Y. Liu, J. Zhao, F. Huang, Y.-B. Cheng, J. Zhong, Printable high-efficiency and stable FAPbBr_3_ perovskite solar cells for multifunctional building-integrated photovoltaics. Adv. Mater. 35, e2301548 (2023). 37219459 10.1002/adma.202301548

[32] T. Bu, J. Li, H. Li, C. Tian, J. Su, G. Tong, L. K. Ono, C. Wang, Z. Lin, N. Chai, X.-L. Zhang, J. Chang, J. Lu, J. Zhong, W. Huang, Y. Qi, Y.-B. Cheng, F. Huang, Lead halide–templated crystallization of methylamine-free perovskite for efficient photovoltaic modules. Science 372, 1327–1332 (2021). 34140385 10.1126/science.abh1035

[33] Y. Zhao, F. Ma, Z. Qu, S. Yu, T. Shen, H.-X. Deng, X. Chu, X. Peng, Y. Yuan, X. Zhang, J. You, Inactive (PbI_2_)_2_RbCl stabilizes perovskite films for efficient solar cells. Science 377, 531–534 (2022). 35901131 10.1126/science.abp8873

[34] Y. Ding, S. Lu, J. Chang, E. Feng, H. Li, C. Long, Y. Yang, C. Yi, Z. Zheng, L. Ding, J. Yang, The molecular additive N-acetyl-L-phenylalanine delays the crystallization and suppresses the phase impurity for achieving triple-cation perovskite solar cells with efficiency over 25%. Small 21, e2410601 (2025). 39580697 10.1002/smll.202410601

[35] H. Xu, Z. Liang, J. Ye, Y. Zhang, Z. Wang, H. Zhang, C. Wan, G. Xu, J. Zeng, B. Xu, Z. Xiao, T. Kirchartz, X. Pan, Constructing robust heterointerfaces for carrier viaduct via interfacial molecular bridges enables efficient and stable inverted perovskite solar cells. Energ. Environ. Sci. 16, 5792–5804 (2023).

[36] Y. Yuan, G. Yan, C. Dreessen, T. Rudolph, M. Hülsbeck, B. Klingebiel, J. Ye, U. Rau, T. Kirchartz, Shallow defects and variable photoluminescence decay times up to 280 μs in triple-cation perovskites. Nat. Mater. 23, 391–397 (2024). 38195863 10.1038/s41563-023-01771-2 PMC10917677

[37] Y.-H. Lin, Vikram, F. Yang, X.-L. Cao, A. Dasgupta, R. D. J. Oliver, A. M. Ulatowski, M. M. McCarthy, X. Shen, Q. Yuan, M. G. Christoforo, F. S. Y. Yeung, M. B. Johnston, N. K. Noel, L. M. Herz, M. S. Islam, H. J. Snaith, Bandgap-universal passivation enables stable perovskite solar cells with low photovoltage loss. Science 384, 767–775 (2024). 38753792 10.1126/science.ado2302

[38] X. Yang, Y.-H. Huang, X.-D. Wang, W.-G. Li, D.-B. Kuang, A-site diamine cation anchoring enables efficient charge transfer and suppressed ion migration in Bi-based hybrid perovskite single crystals. Angew. Chem. Int. Ed. Engl. 61, e202204663 (2022). 35527378 10.1002/anie.202204663

[39] G. Yang, Z. Ni, Z. J. Yu, B. W. Larson, Z. Yu, B. Chen, A. Alasfour, X. Xiao, J. M. Luther, Z. C. Holman, J. Huang, Defect engineering in wide-bandgap perovskites for efficient perovskite–silicon tandem solar cells. Nat. Photonics 16, 588–594 (2022).

[40] F. Li, X. Deng, F. Qi, Z. Li, D. Liu, D. Shen, M. Qin, S. Wu, F. Lin, S.-H. Jang, J. Zhang, X. Lu, D. Lei, C.-S. Lee, Z. Zhu, A. K. Y. Jen, Regulating surface termination for efficient inverted perovskite solar cells with greater than 23% efficiency. J. Am. Chem. Soc. 142, 20134–20142 (2020). 33190487 10.1021/jacs.0c09845

[41] T. Ma, H. Wang, Z. Wu, Y. Zhao, C. Chen, X. Yin, L. Hu, F. Yao, Q. Lin, S. Wang, D. Zhao, X. Li, C. Wang, Hole transport layer-free low-bandgap perovskite solar cells for efficient all-perovskite tandems. Adv. Mater. 36, 2308240 (2024). 10.1002/adma.202308240 37967309

[42] Z. Wu, Y. Zhao, C. Wang, T. Ma, C. Chen, Y. Liu, T. Jia, Y. Zhai, C. Chen, C. Zhang, G. Cao, Z. Yang, D. Zhao, X. Li, Enhancing photovoltaically preferred orientation in wide-bandgap perovskite for efficient all-perovskite tandem solar cells. Adv. Mater. 37, e2412943 (2025). 39763405 10.1002/adma.202412943

[43] C. Jiang, T. Qin, L. Tan, H. Li, J. Zhou, M. Li, Z.-M. Dang, L. Ding, Q. Xiong, C. Yi, Revealing the hole and electron transport dynamics in the working devices for efficient semitransparent perovskite solar cells. Adv. Energy Mater. 14, 2304093 (2024).

[44] Q. Jiang, J. Tong, Y. Xian, R. A. Kerner, S. P. Dunfield, C. Xiao, R. A. Scheidt, D. Kuciauskas, X. Wang, M. P. Hautzinger, R. Tirawat, M. C. Beard, D. P. Fenning, J. J. Berry, B. W. Larson, Y. Yan, K. Zhu, Surface reaction for efficient and stable inverted perovskite solar cells. Nature 611, 278–283 (2022). 36049505 10.1038/s41586-022-05268-x

[45] S. Qin, C. Lu, Z. Jia, Y. Wang, S. Li, W. Lai, P. Shi, R. Wang, C. Zhu, J. Du, J. Zhang, L. Meng, Y. Li, Constructing monolithic perovskite/organic tandem solar cell with efficiency of 22.0% via reduced open-circuit voltage loss and broadened absorption spectra. Adv. Mater. 34, e2108829 (2022). 35048434 10.1002/adma.202108829

[46] S. Li, Z. Zheng, J. Ju, S. Cheng, F. Chen, Z. Xue, L. Ma, Z. Wang, A generic strategy to stabilize wide bandgap perovskites for efficient tandem solar cells. Adv. Mater. 36, 2307701 (2024). 10.1002/adma.202307701 38061761

[47] Z. Zhang, J. Wang, J. Liang, Y. Zheng, X. Wu, C. Tian, A. Sun, Y. Huang, Z. Zhou, Y. Yang, Y. Liu, C. Tang, Z. Chen, C.-C. Chen, Organizing uniform phase distribution in methylammonium-free 1.77 eV wide-bandgap inverted perovskite solar cells. Small 19, e2303213 (2023). 37269195 10.1002/smll.202303213

[48] A. Zhang, M. Li, C. Dong, W. Ye, X. Yang, A. Shaker, M. S. Salem, Z. Li, J. Yang, X. Li, L. Xu, H. Song, C. Chen, J. Tang, π–π stacking at the perovskite/C_60_ interface enables high-efficiency wide-bandgap perovskite solar cells. Small 20, 2401197 (2024). 10.1002/smll.202401197 38676332

[49] Y. Luo, J. Zhu, X. Yin, W. Jiao, Z. Gao, Y. Xu, C. Wang, Y. Wang, H. Lai, H. Huang, J. Luo, J. Wang, J. You, Z. Zhang, X. Hao, G. Zeng, S. Ren, Z. Li, F. Fu, M. Li, C. Xiao, C. Chen, D. Zhao, Enhanced efficiency and stability of wide-bandgap perovskite solar cells via molecular modification with piperazinium salt. Adv. Energy Mater. 14, 2304429 (2024).

[50] X. Shen, B. M. Gallant, P. Holzhey, J. A. Smith, K. A. Elmestekawy, Z. Yuan, P. V. G. M. Rathnayake, S. Bernardi, A. Dasgupta, E. Kasparavicius, T. Malinauskas, P. Caprioglio, O. Shargaieva, Y.-H. Lin, M. M. McCarthy, E. Unger, V. Getautis, A. Widmer-Cooper, L. M. Herz, H. J. Snaith, Chloride-based additive engineering for efficient and stable wide-bandgap perovskite solar cells. Adv. Mater. 35, e2211742 (2023). 37191054 10.1002/adma.202211742

[51] G. Xie, H. Li, X. Wang, J. Fang, D. Lin, D. Wang, S. Li, S. He, L. Qiu, Phase segregation and voltage loss mitigated highly efficient perovskite–organic tandem solar cells with a simple ambipolar SnO*_X_* interconnecting layer. Adv. Funct. Mater. 33, 2308794 (2023).

[52] Z. Yi, W. Wang, R. He, J. Zhu, W. Jiao, Y. Luo, Y. Xu, Y. Wang, Z. Zeng, K. Wei, J. Zhang, S.-W. Tsang, C. Chen, W. Tang, D. Zhao, Achieving a high open-circuit voltage of 1.339 V in 1.77 eV wide-bandgap perovskite solar cells via self-assembled monolayers. Energ. Environ. Sci. 17, 202–209 (2024).

[53] P. Chen, Y. Xiao, J. Hu, S. Li, D. Luo, R. Su, P. Caprioglio, P. Kaienburg, X. Jia, N. Chen, J. Wu, Y. Sui, P. Tang, H. Yan, T. Huang, M. Yu, Q. Li, L. Zhao, C.-H. Hou, Y.-W. You, J.-J. Shyue, D. Wang, X. Li, Q. Zhao, Q. Gong, Z.-H. Lu, H. J. Snaith, R. Zhu, Multifunctional ytterbium oxide buffer for perovskite solar cells. Nature 625, 516–522 (2024). 38233617 10.1038/s41586-023-06892-x

[54] J. Wen, Y. Zhao, P. Wu, Y. Liu, X. Zheng, R. Lin, S. Wan, K. Li, H. Luo, Y. Tian, L. Li, H. Tan, Heterojunction formed via 3D-to-2D perovskite conversion for photostable wide-bandgap perovskite solar cells. Nat. Commun. 14, 7118 (2023). 37932289 10.1038/s41467-023-43016-5 PMC10628126

[55] H. Cui, L. Huang, S. Zhou, C. Wang, X. Hu, H. Guan, S. Wang, W. Shao, D. Pu, K. Dong, J. Zhou, P. Jia, W. Wang, C. Tao, W. Ke, G. Fang, Lead halide coordination competition at buried interfaces for low V_OC_-deficits in wide-bandgap perovskite solar cells. Energ. Environ. Sci. 16, 5992–6002 (2023).

[56] J. Zhou, T. Wen, J. Sun, Z. Shi, C. Zou, Z. Shen, Y. Li, Y. Wang, Y. Lin, S. Yang, F. Liu, Z. Yang, Phase-stable wide-bandgap perovskites with 2D/3D structure for all-perovskite tandem solar cells. ACS Energy Lett. 9, 1984–1992 (2024).

[57] H. Chen, A. Maxwell, C. Li, S. Teale, B. Chen, T. Zhu, E. Ugur, G. Harrison, L. Grater, J. Wang, Z. Wang, L. Zeng, S. M. Park, L. Chen, P. Serles, R. A. Awni, B. Subedi, X. Zheng, C. Xiao, N. J. Podraza, T. Filleter, C. Liu, Y. Yang, J. M. Luther, S. De Wolf, M. G. Kanatzidis, Y. Yan, E. H. Sargent, Regulating surface potential maximizes voltage in all-perovskite tandems. Nature 613, 676–681 (2023). 36379225 10.1038/s41586-022-05541-z

[58] S. Tan, C. Li, C. Peng, W. Yan, H. Bu, H. Jiang, F. Yue, L. Zhang, H. Gao, Z. Zhou, Sustainable thermal regulation improves stability and efficiency in all-perovskite tandem solar cells. Nat. Commun. 15, 4136 (2024). 38755156 10.1038/s41467-024-48552-2 PMC11099067

[59] Y. Bai, R. Tian, K. Sun, C. Liu, X. Lang, M. Yang, Y. Meng, C. Xiao, Y. Wang, X. Lu, J. Wang, H. Pan, Z. Song, S. Zhou, Z. Ge, Decoupling light-and oxygen-induced degradation mechanisms of Sn–Pb perovskites in all perovskite tandem solar cells. Energ. Environ. Sci. 17, 8557–8569 (2024).

[60] J. Zhu, Y. Xu, Y. Luo, J. Luo, R. He, C. Wang, Y. Wang, K. Wei, Z. Yi, Z. Gao, J. Wang, J. You, Z. Zhang, H. Lai, S. Ren, X. Liu, C. Xiao, C. Chen, J. Zhang, F. Fu, D. Zhao, Custom-tailored hole transport layer using oxalic acid for high-quality tin-lead perovskites and efficient all-perovskite tandems. Sci. Adv. 10, eadl2063 (2024). 38640232 10.1126/sciadv.adl2063 PMC11029806

[61] Y. Pan, J. Wang, Z. Sun, J. Zhang, Z. Zhou, C. Shi, S. Liu, F. Ren, R. Chen, Y. Cai, H. Sun, B. Liu, Z. Zhang, Z. Zhao, Z. Cai, X. Qin, Z. Zhao, Y. Ji, N. Li, W. Huang, Z. Liu, W. Chen, Surface chemical polishing and passivation minimize non-radiative recombination for all-perovskite tandem solar cells. Nat. Commun. 15, 7335 (2024). 39187539 10.1038/s41467-024-51703-0 PMC11347601

[62] D. Yu, M. Pan, G. Liu, X. Jiang, X. Wen, W. Li, S. Chen, W. Zhou, H. Wang, Y. Lu, M. Ma, Z. Zang, P. Cheng, Q. Ji, F. Zheng, Z. Ning, Electron-withdrawing organic ligand for high-efficiency all-perovskite tandem solar cells. Nat. Energy 9, 298–307 (2024).

[63] W. Shen, H. Fang, D. Pu, W. Zheng, X. Zhang, G. Li, L. Huang, S. Zhou, W. Chen, Y. Zhou, Z. Feng, J. Liang, J. Zhou, P. Qin, G. Fang, W. Ke, Optimizing blade-coated tin–lead perovskite solar cells and tandems with multi-carboxyl and amino group integration. Adv. Funct. Mater. 34, 2410605 (2024).

[64] G. Liu, G. Yang, W. Feng, H. Li, M. Yang, Y. Zhong, X. Jiang, W.-Q. Wu, Regulating surface metal abundance via lattice-matched coordination for versatile and environmentally-viable Sn-Pb alloying perovskite solar cells. Adv. Mater. 36, 2405860 (2024). 10.1002/adma.202405860 39108194

[65] X. Jiang, Q. Zhou, Y. Lu, H. Liang, W. Li, Q. Wei, M. Pan, X. Wen, X. Wang, W. Zhou, D. Yu, H. Wang, N. Yin, H. Chen, H. Li, T. Pan, M. Ma, G. Liu, W. Zhou, Z. Su, Q. Chen, F. Fan, F. Zheng, X. Gao, Q. Ji, Z. Ning, Surface heterojunction based on n-type low-dimensional perovskite film for highly efficient perovskite tandem solar cells. Nat. Sci. Rev. 11, nwae055 (2024). 10.1093/nsr/nwae055 PMC10989298 38577668

[66] G. Li, C. Wang, S. Fu, W. Zheng, W. Shen, P. Jia, L. Huang, S. Zhou, J. Zhou, C. Wang, H. Guan, Y. Zhou, X. Zhang, D. Pu, H. Fang, Q. Lin, W. Ai, W. Chen, G. Zeng, T. Wang, P. Qin, G. Fang, W. Ke, Boosting all-perovskite tandem solar cells by revitalizing the buried tin-lead perovskite interface. Adv. Mater. 36, e2401698 (2024). 39075821 10.1002/adma.202401698

[67] Q. Sun, Z. Zhang, H. Yu, J. Huang, X. Li, L. Dai, Q. Wang, Y. Shen, M. Wang, Surface charge transfer doping of narrow-bandgap Sn–Pb perovskites for high-performance tandem solar cells. Energ. Environ. Sci. 17, 2512–2520 (2024).

[68] J. Wang, Y. Pan, Z. Zhou, Q. Zhou, S. Liu, J. Zhang, C. Shi, R. Chen, Z. Zhao, Z. Cai, X. Qin, Z. Zhao, Z. Yang, Z. Liu, W. Chen, Bimolecular crystallization modulation boosts the efficiency and stability of methylammonium-free tin–lead perovskite and all-perovskite tandem solar cells. Adv. Energy Mater. 14, 2402171 (2024).

[69] Y.-H. Chiang, K. Frohna, H. Salway, A. Abfalterer, L. Pan, B. Roose, M. Anaya, S. D. Stranks, Vacuum-deposited wide-bandgap perovskite for all-perovskite tandem solar cells. ACS Energy Lett. 8, 2728–2737 (2023). 37324541 10.1021/acsenergylett.3c00564 PMC10262197

[70] J. Zhou, H. Qiu, T. Wen, Z. He, C. Zou, Y. Shi, L. Zhu, C.-C. Chen, G. Liu, S. Yang, F. Liu, Z. Yang, Acidity control of interface for improving stability of all-perovskite tandem solar cells. Adv. Energy Mater. 13, 2300968 (2023).

[71] J. Wen, Y. Zhao, Z. Liu, H. Gao, R. Lin, S. Wan, C. Ji, K. Xiao, Y. Gao, Y. Tian, J. Xie, C. J. Brabec, H. Tan, Steric engineering enables efficient and photostable wide-bandgap perovskites for all-perovskite tandem solar cells. Adv. Mater. 34, e2110356 (2022). 35439839 10.1002/adma.202110356

[72] B. Chen, Z. Yu, A. Onno, Z. Yu, S. Chen, J. Wang, Z. C. Holman, J. Huang, Bifacial all-perovskite tandem solar cells. Sci. Adv. 8, eadd0377 (2022). 36427306 10.1126/sciadv.add0377 PMC9699687

[73] R. Prasanna, T. Leijtens, S. P. Dunfield, J. A. Raiford, E. J. Wolf, S. A. Swifter, J. Werner, G. E. Eperon, C. de Paula, A. F. Palmstrom, C. C. Boyd, M. F. A. M. van Hest, S. F. Bent, G. Teeter, J. J. Berry, M. D. McGehee, Design of low bandgap tin–lead halide perovskite solar cells to achieve thermal, atmospheric and operational stability. Nat. Energy 4, 939–947 (2019).

[74] A. F. Palmstrom, G. E. Eperon, T. Leijtens, R. Prasanna, S. N. Habisreutinger, W. Nemeth, E. A. Gaulding, S. P. Dunfield, M. Reese, S. Nanayakkara, T. Moot, J. Werner, J. Liu, B. To, S. T. Christensen, M. D. McGehee, M. F. A. M. van Hest, J. M. Luther, J. J. Berry, D. T. Moore, Enabling flexible all-perovskite tandem solar cells. Joule 3, 2193–2204 (2019).

