# Supplementary material for: Defect passivation and crystallization modulation in methylammonium-free wide-bandgap perovskites for all-perovskite tandem solar cells
Source: Sci Adv. 2025 Sep 17;11(38):eadv4501. doi: 10.1126/sciadv.adv4501 (PMC12442863; doi:10.1126/sciadv.adv4501)
Supplement: Supplementary file 1 — Figs. S1 to S34 Tables S1 to S13 References [file sciadv.adv4501_sm.pdf]

Science Advances
AAAS

Supplementary Materials for

Defect passivation and crystallization modulation in methylammonium-free wide-bandgap perovskites for all-perovskite tandem solar cells

Xuefei Jia *et al.*

Corresponding author: Jinhui Tong, jinhui.tong@whut.edu.cn; Yaxin Zhai, yzhai@hunnu.edu.cn

Sci. Adv. **11**, eadv4501 (2025)
DOI: 10.1126/sciadv.adv4501

This PDF file includes:

Figs. S1 to S34
Tables S1 to S13
References

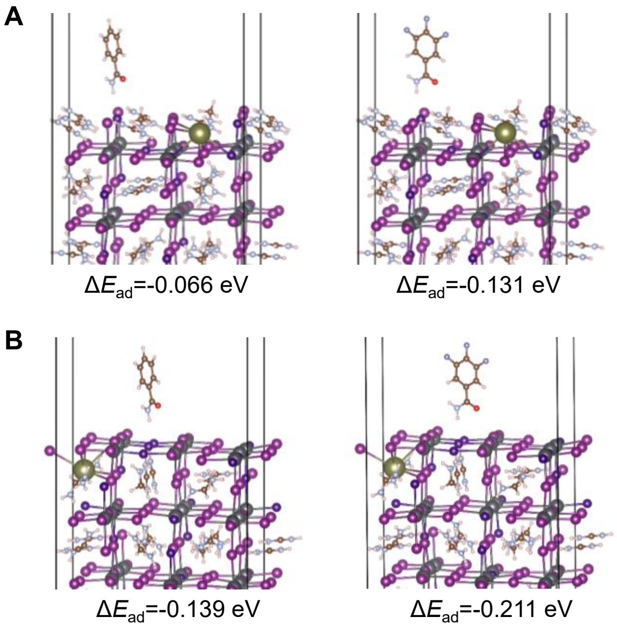

Fig. S1. DFT calculations of adsorption energies. (A) DFT calculations of the adsorption energies of BZD/TFBZ molecules on the FABr termination. (B) DFT calculations of the adsorption energies of BZD/TFBZ molecules on the $PbBr_2$ termination.

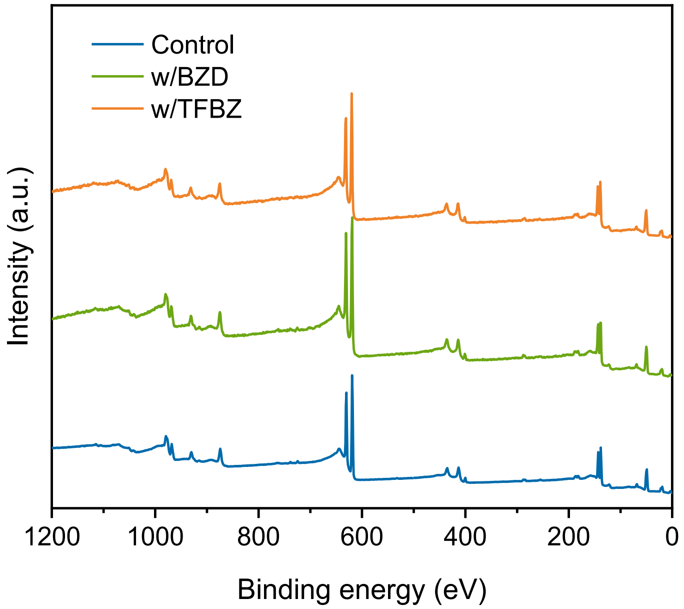

Fig. S2. XPS spectra of the control, w/BZD, and w/TFBZ perovskite films.

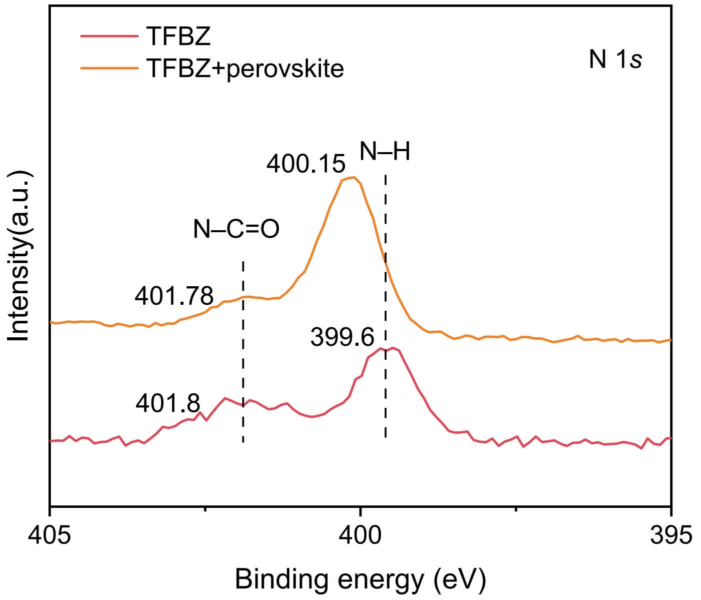

Fig. S3. XPS spectra of N 1*s* for the TFBZ and its mixture with perovskite.

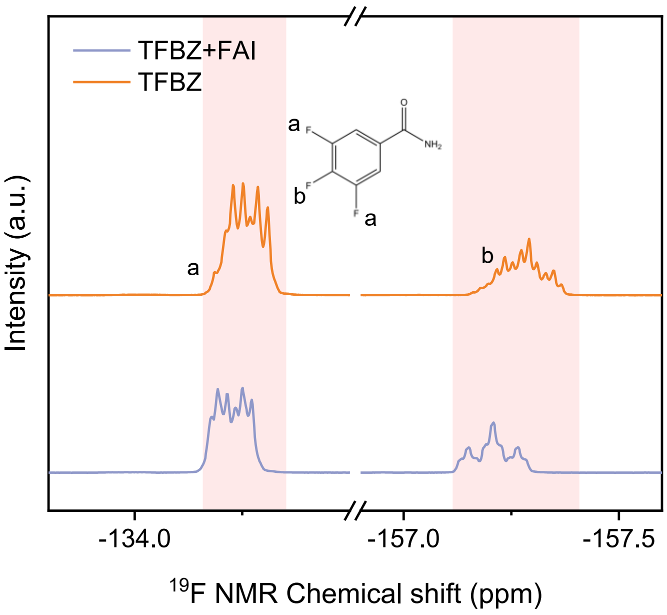

Fig. S4. ^{19}F NMR spectra of TFBZ and its mixture with FAI in DMSO-*d*$_6$ solvent.

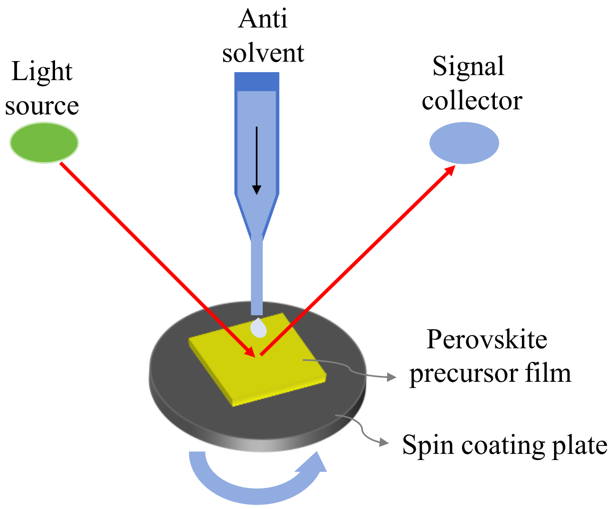

Fig. S5. Schematic of the *in situ* UV-vis transmission measurement consisting of the light source and signal collector.

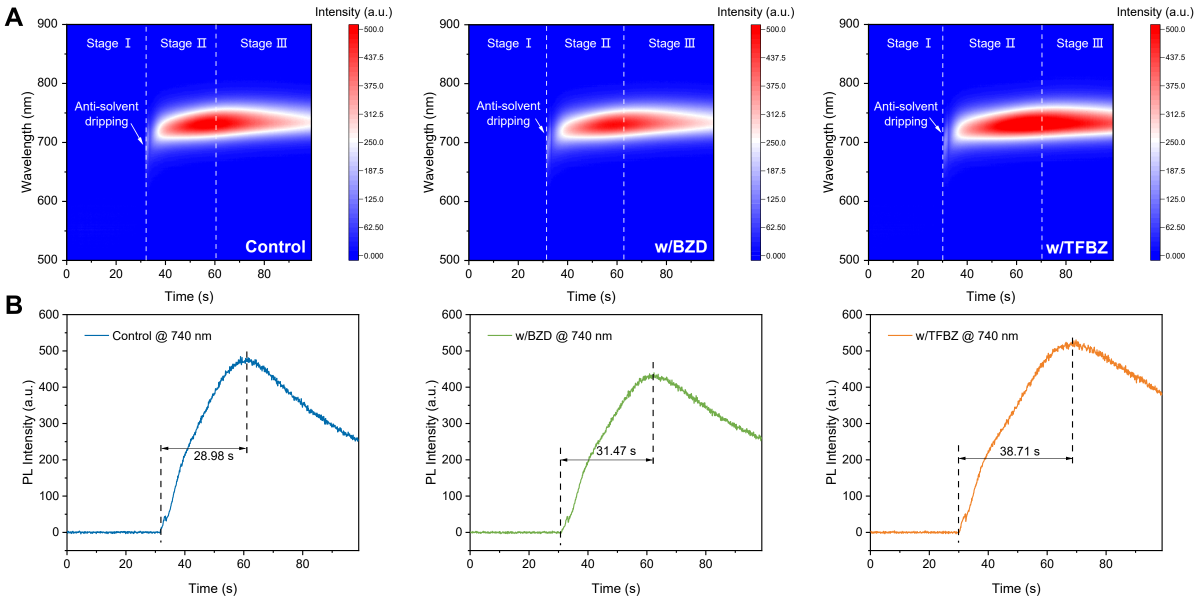

Fig. S6. ***In situ*** **PL spectra of perovskite films. (A)** 2D contour maps of PL spectra for the control, w/BZD, and w/TFBZ films during the spin coating process. **(B)** The dynamic evolution of PL intensity of control, w/BZD, and w/TFBZ films at 740 nm during the spin coating process.

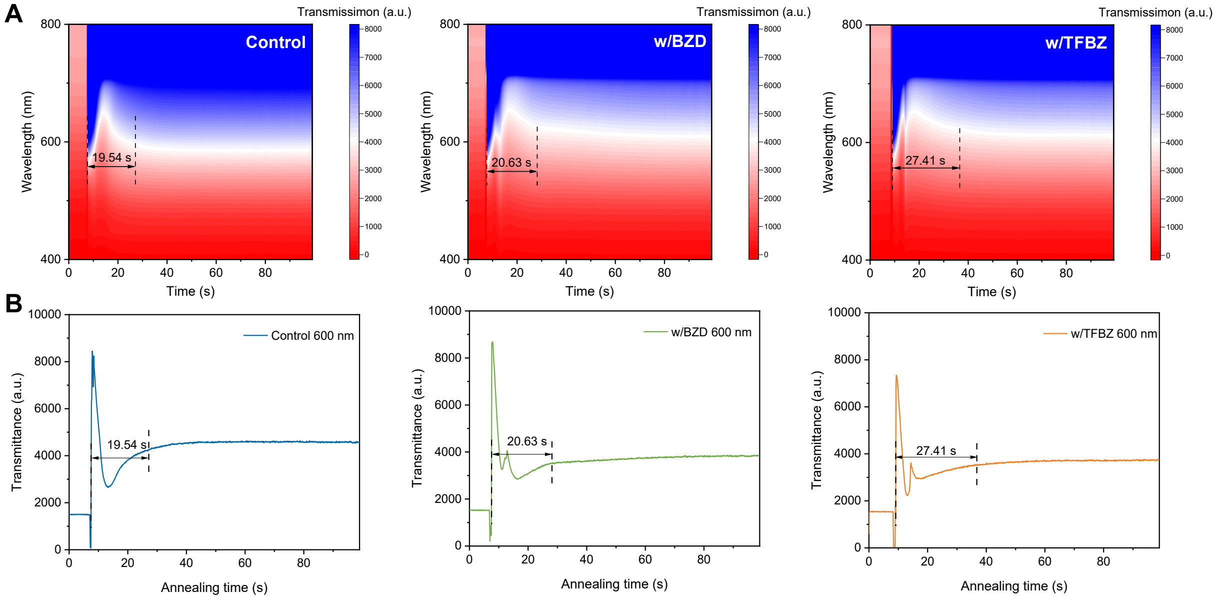

Fig. S7. ***In situ*** **UV-vis transmission of perovskite films. (A)** 2D contour maps of the dynamic evolution of UV-vis transmission of control, w/BZD, and w/TFBZ films during the annealing process. **(B)** Dynamic evolution of transmission at 600 nm of control, w/BZD, and w/TFBZ films during the annealing process.

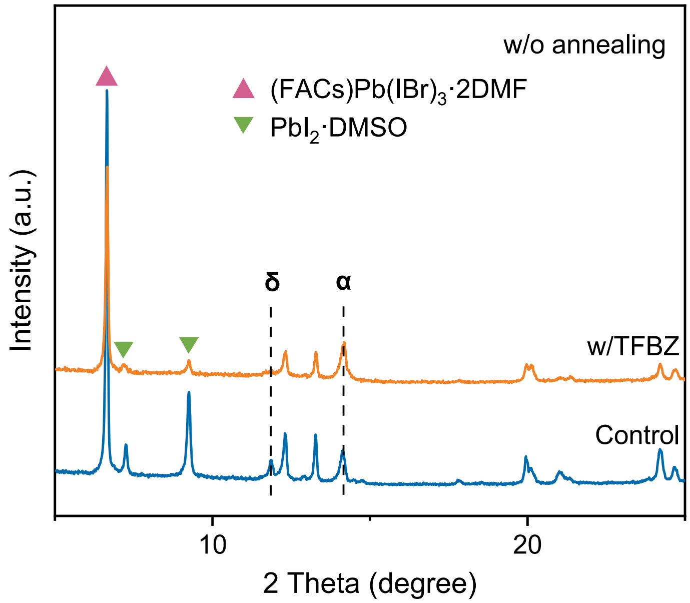

Fig. S8. XRD pattern of unannealed control, w/BZD, and w/TFBZ wet perovskite films.

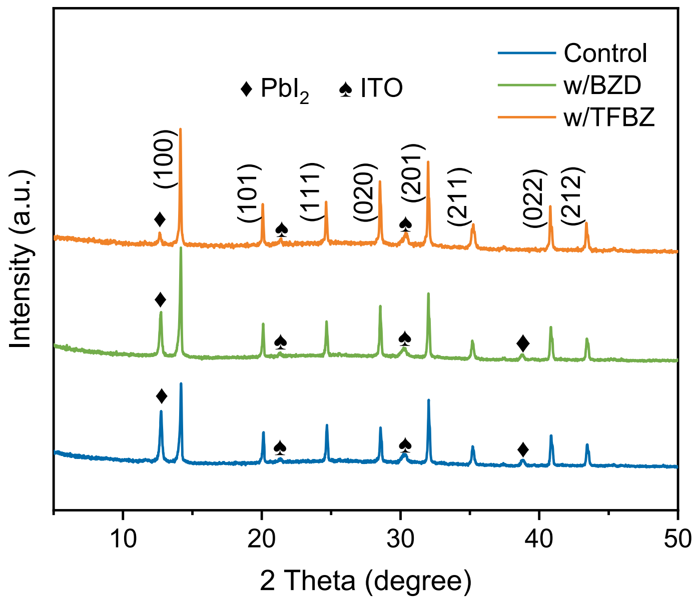

Fig. S9. XRD pattern of control, w/BZD, and w/TFBZ perovskite films.

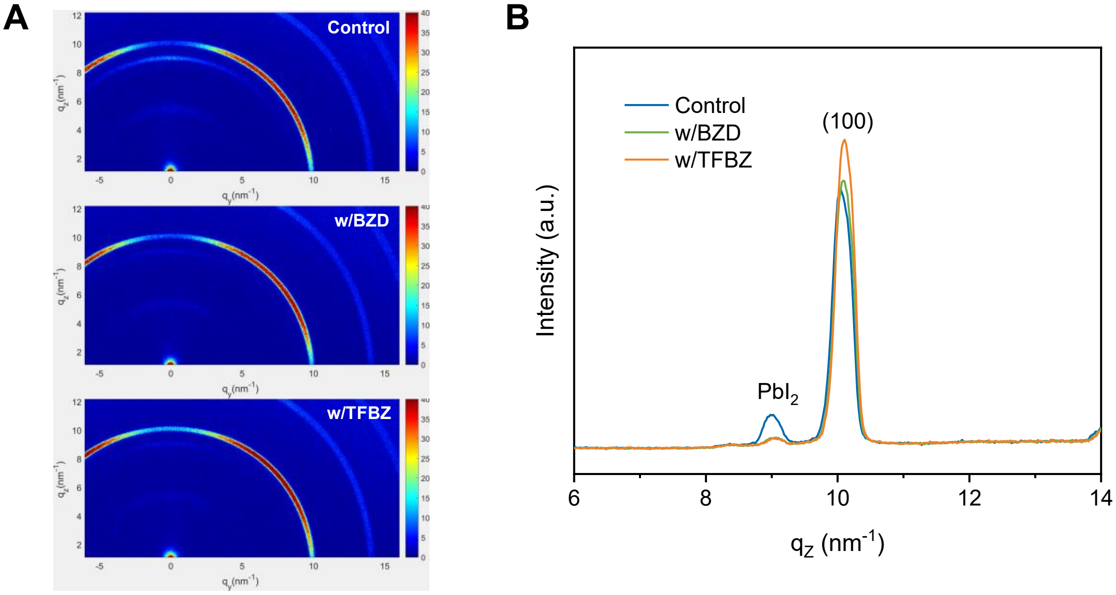

Fig. S10. GIWAXS study on additive-induced crystallization modification. (A) 2D GIWAXS pattern of the control, w/BZD, and w/TFBZ perovskite films. **(B)** 1D out-of-plane radial cake cut profiles of the control, w/BZD, and w/TFBZ perovskite films.

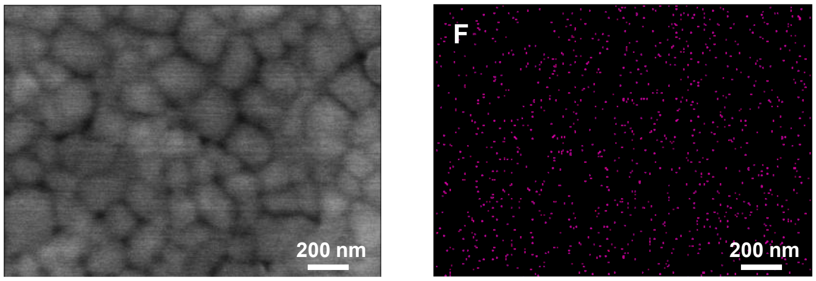

Fig. S11. SEM image and corresponding EDS elemental mapping of the TFBZ-based perovskite surface.

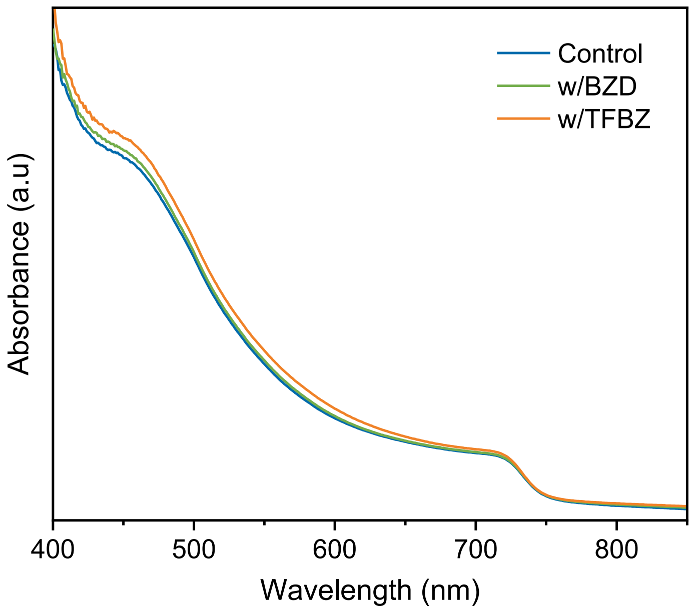

Fig. S12. UV-vis absorption spectra of the control, w/BZD, and w/TFBZ perovskite films.

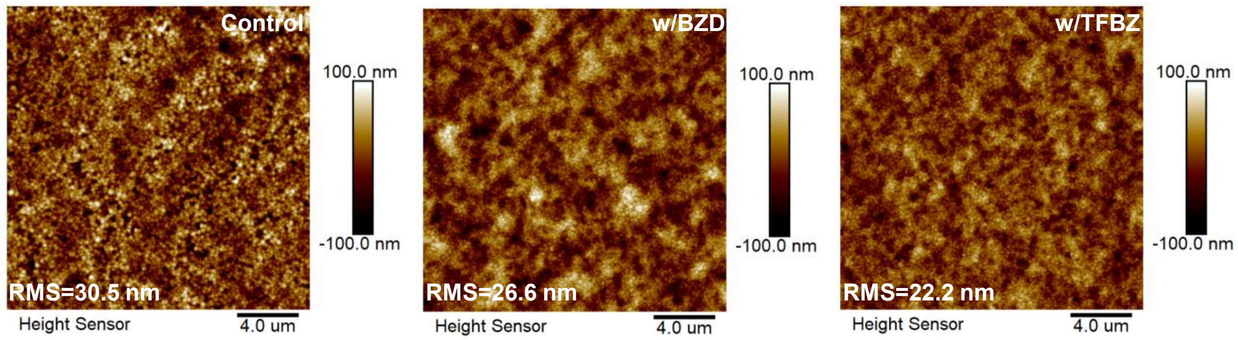

Fig. S13. AFM images of control, w/BZD, and w/TFBZ perovskite films.

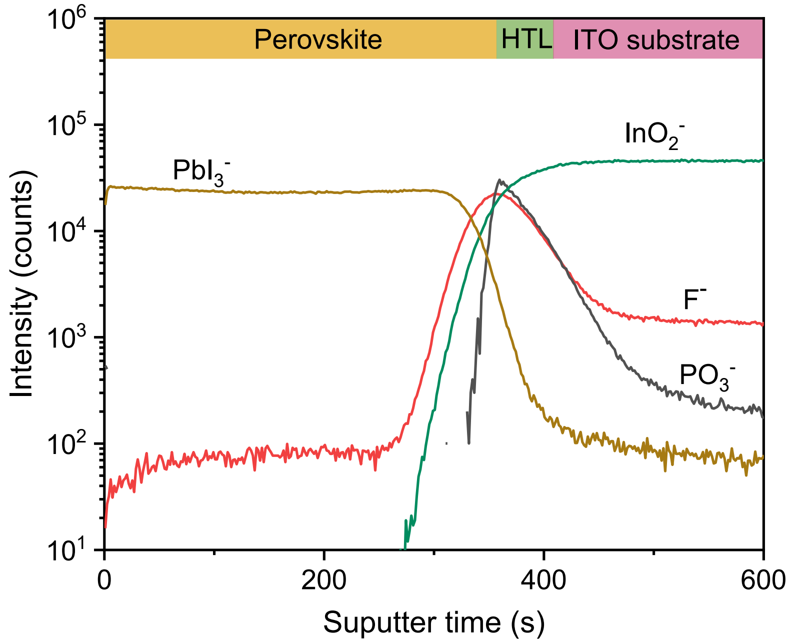

Fig. S14. TOF-SIMS depth profile of w/TFBZ perovskite film.

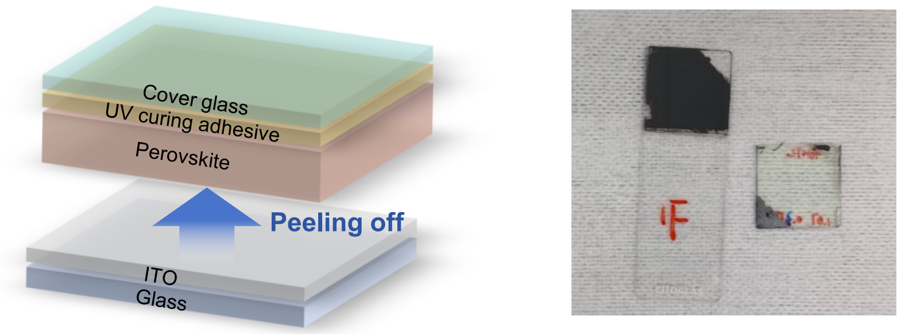

Fig. S15. Schematic diagram of the preparation process and photography of the bottom of perovskite films. The bottom surface sample of perovskite film was fabricated with the following method. Firstly, spinning coat the perovskite onto a clean ITO. Stick glass slides and perovskite film surfaces together using UV glue. After the glue was solidified, the film was peeled off from the ITO, and then the bottom surface of the perovskite film for characterization was obtained.

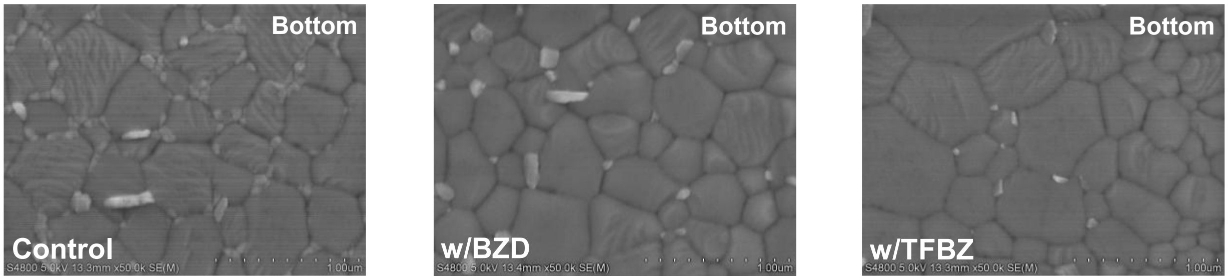

Fig. S16. SEM images of control, w/BZD, and w/TFBZ perovskite buried surfaces.

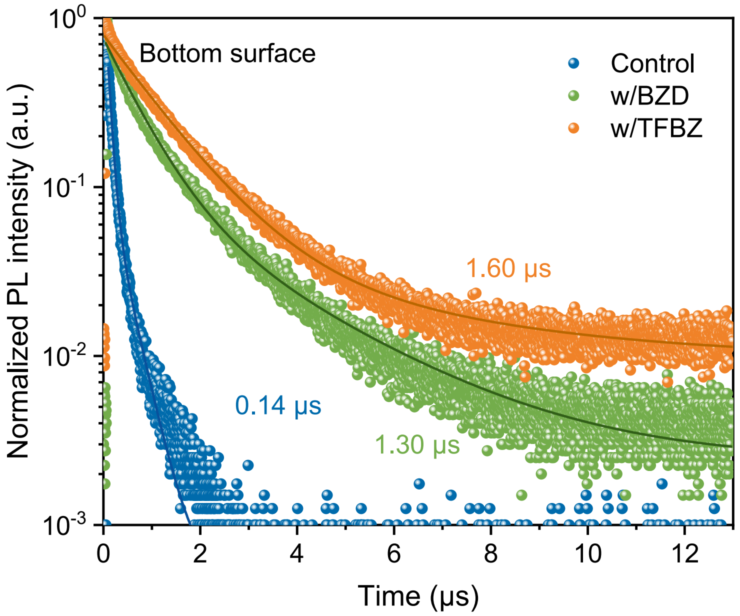

Fig. S17. TRPL spectra detected on the bottom surface of the control, w/BZD, and w/TFBZ perovskite films.

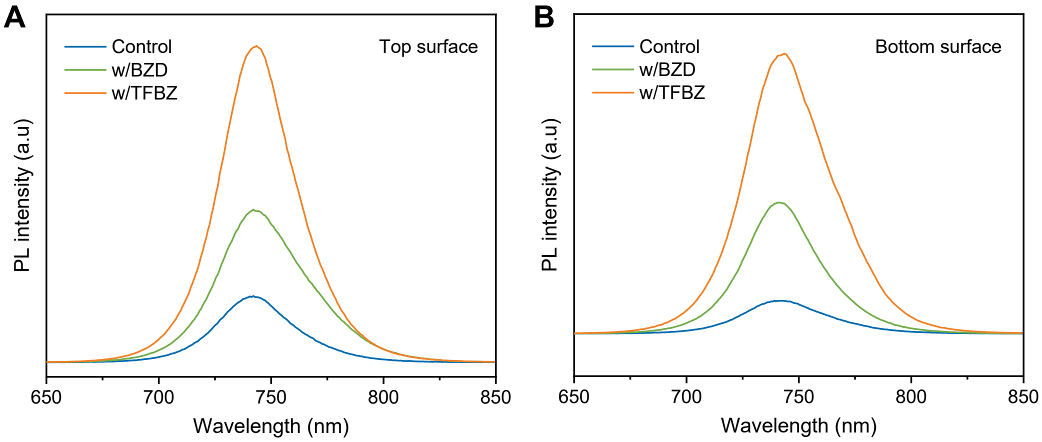

Fig. S18. PL spectra detected on (A) the top surface and (B) the bottom surface of control, w/BZD, and w/TFBZ perovskite films.

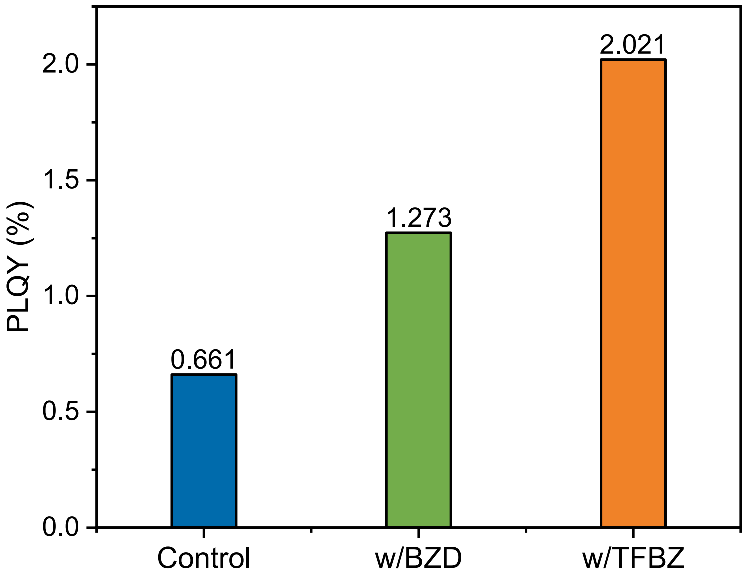

Fig. S19. PLQY of the control, w/BZD, and w/TFBZ perovskite films deposited on glass.

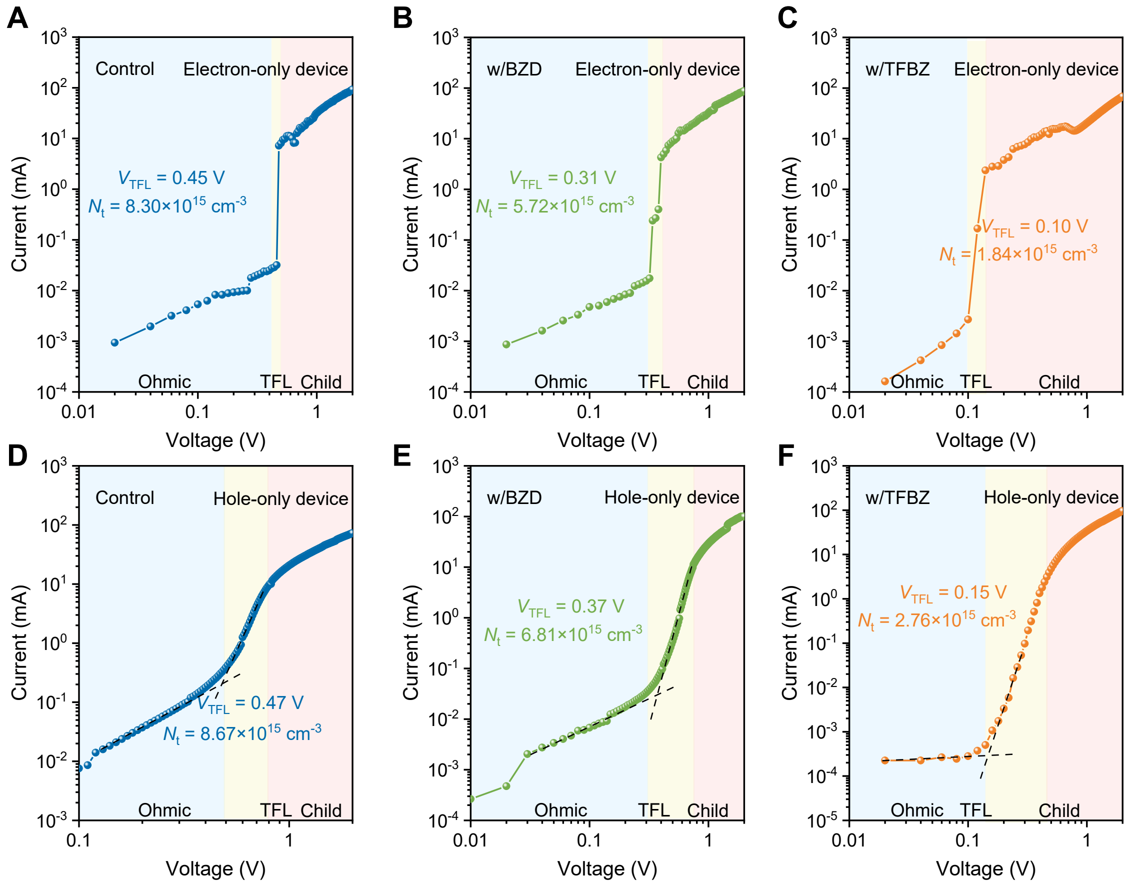

Fig. S20. SCLC measurements of electron-only devices (ITO/C_{60}/perovskite /C_{60}/Ag) for (A) control, (B) w/BZD, and (C) w/TFBZ films and hole-only devices (ITO/ Me-4PACz/perovskite /PTAA/Ag) for (D) control, (E) w/BZD, and (F) w/TFBZ films.

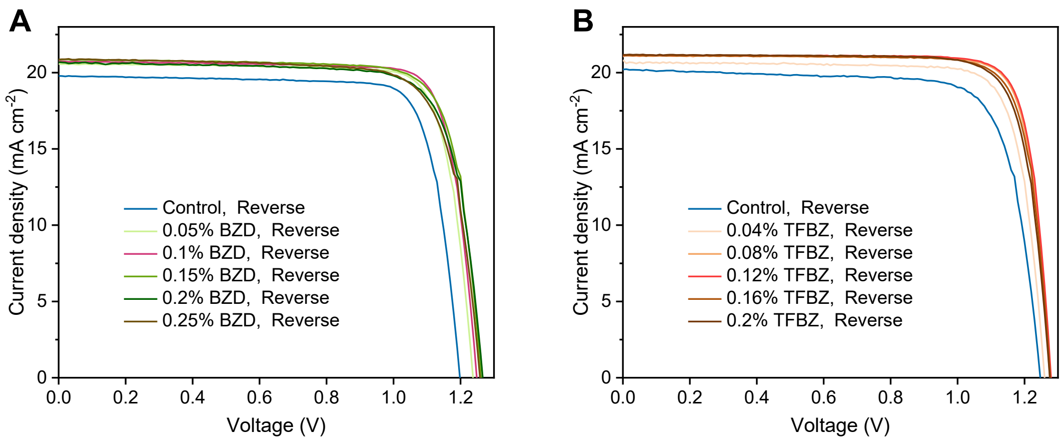

Fig. S21. ***J-V*** **curves of 1.67 eV WBG PSCs with different contents of (A) BZD and (B) TFBZ.**

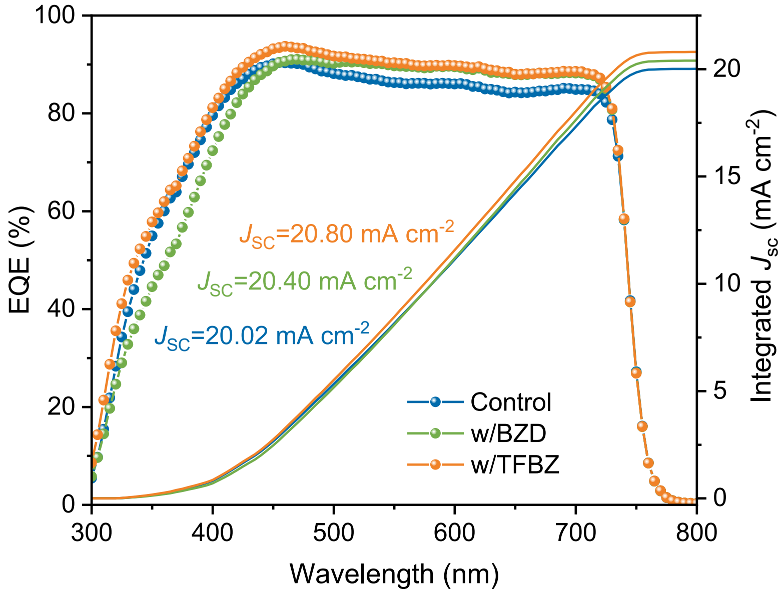

Fig. S22. EQE and integrated current density spectra of 1.67 eV WBG devices for control, w/BZD, and w/TFBZ.

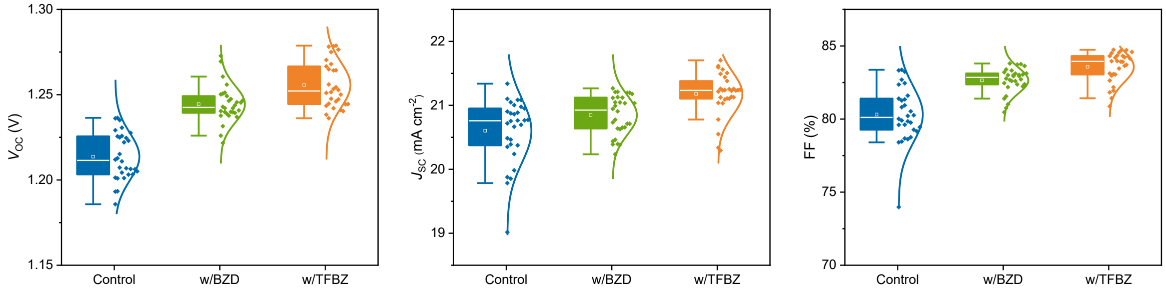

Fig. S23. Statistical analysis of photovoltaic parameters for 1.67 eV WBG PSCs with and without additives.

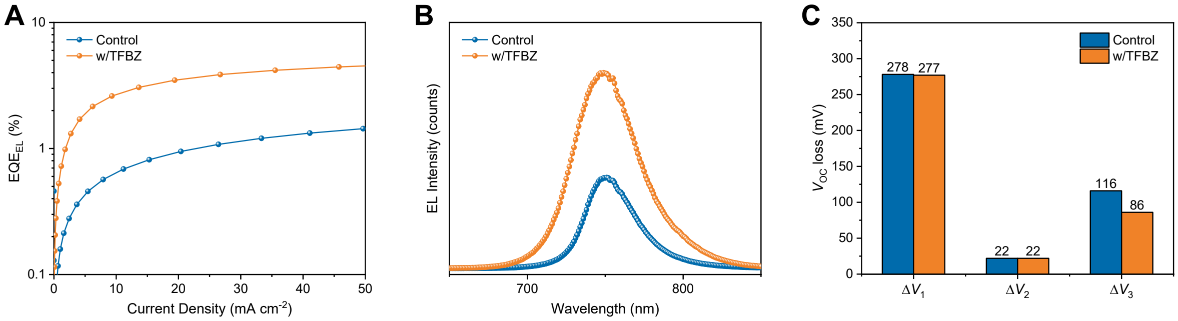

Fig. S24. Electroluminescence behavior and V_{OC} losses in the 1.67 eV WBG devices without and with TFBZ. (A) EQE_{EL} spectra of the PSCs working as LEDs under different voltages. **(B)** EL spectra. **(C)** V_{OC} loss analysis of the corresponding devices.

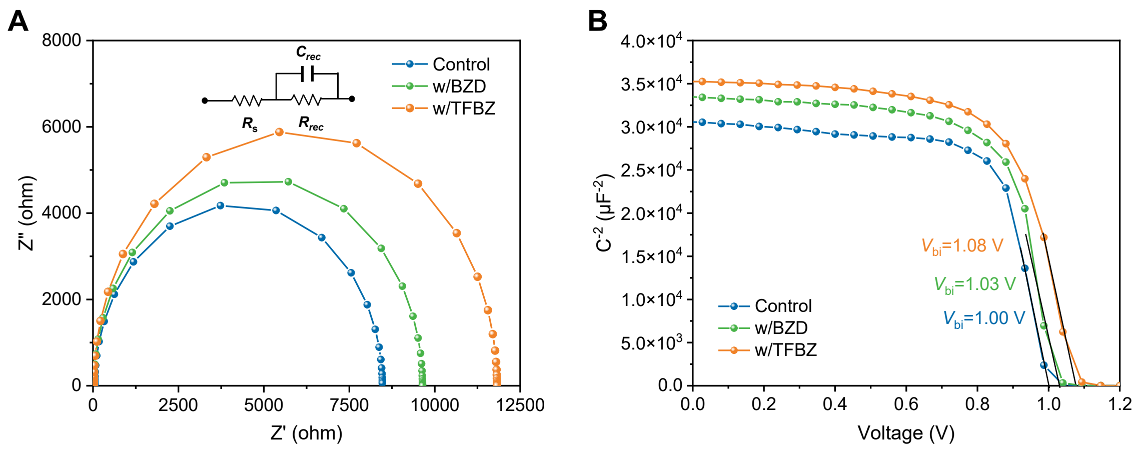

Fig. S25. Electrochemical impedance and Mott-Schottky study of the 1.67 eV WBG devices without and with additives. (A) Nyquist plots. **(B)** Mott-Schottky curves.

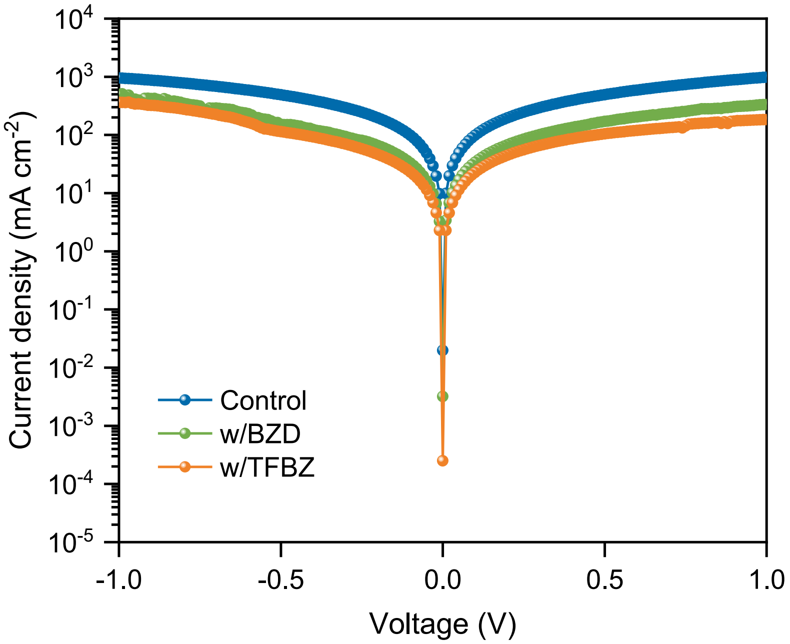

Fig. S26. Dark *J-V* curves for the 1.67 eV WBG devices without and with additives.

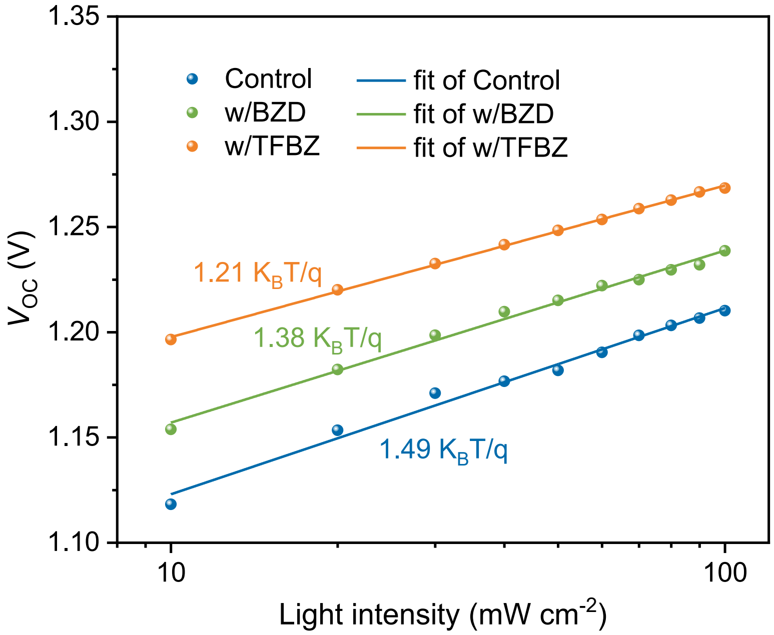

Fig. S27. Light intensity dependence of V_{OC} and slopes of the linear fits for the 1.67 eV WBG devices without and with additives.

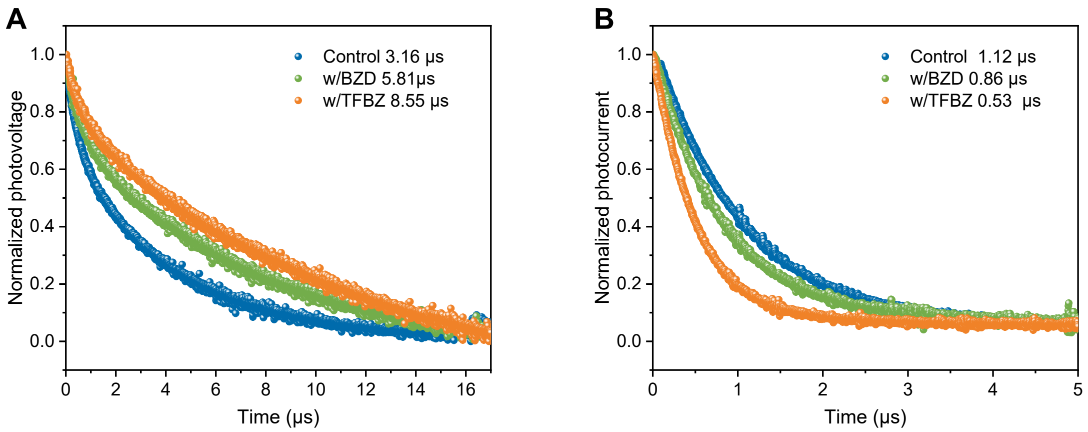

Fig. S28. Charge carrier transport and extraction kinetics of 1.67 eV WBG devices without and with additives. (A) Normalized TPV decay. **(B)** Normalized TPC decay.

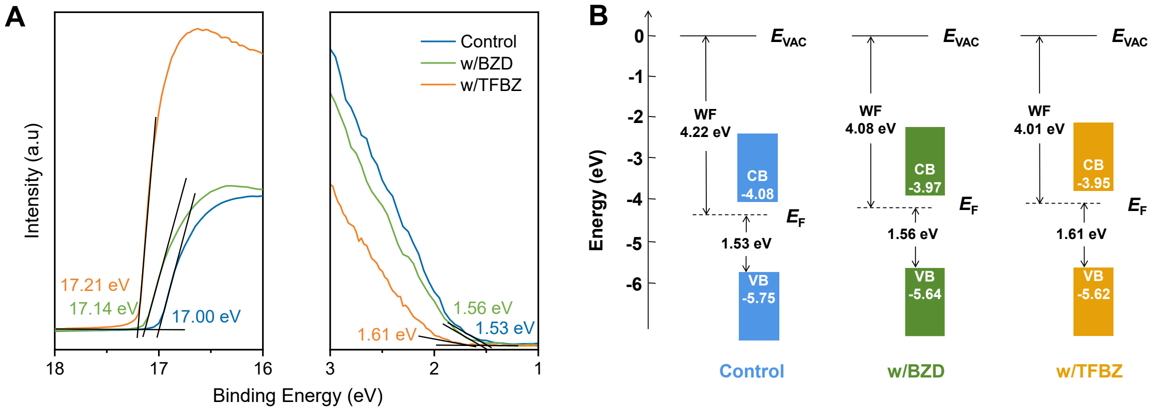

Fig. S29. Energetics of the 1.67 eV WBG perovskite films. (A) UPS energy spectra of control, w/BZD, and w/TFBZ perovskite films. **(B)** Energy level diagram of the control, w/BZD, and w/TFBZ perovskite films.

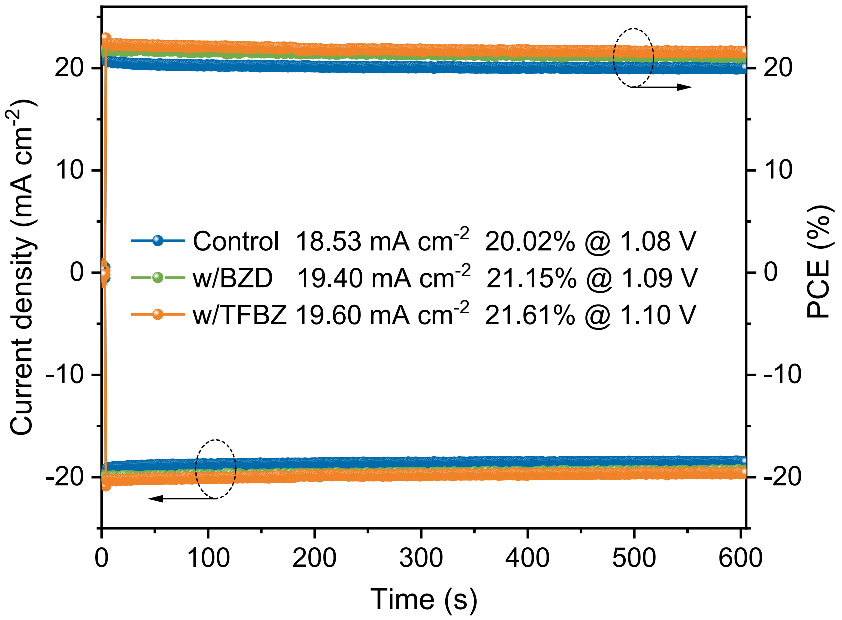

Fig. S30. Current density and SPO measured for 600 s at a fixed voltage near MPP identified in the *J-V* curves of the 1.67 eV WBG devices without and with additives.

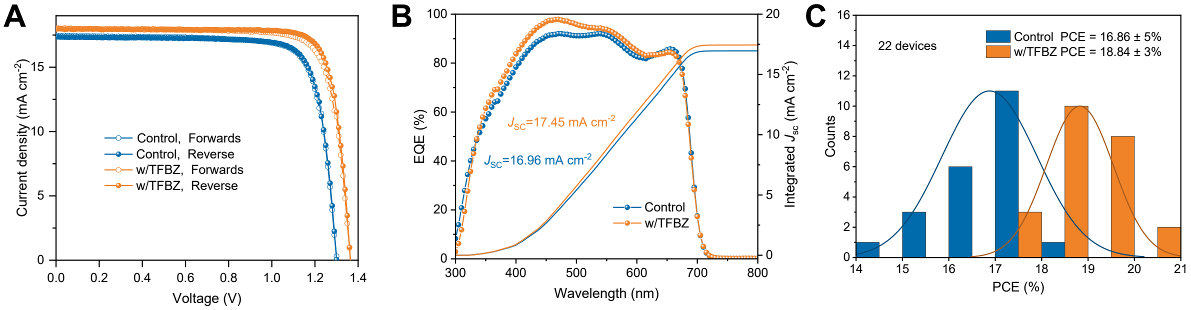

Fig. S31. Photovoltaic parameters of the 1.79 eV WBG PSCs without and with TFBZ. (A) *J*-*V* curves. **(B)** EQE curves. **(C)** histograms of PCE distributions.

中国认可
国际互认
检测
TESTING
CNAS L8490
230017163974

Test and Calibration Center of New Energy Device and Module,
Shanghai Institute of Microsystem and Information Technology,
Chinese Academy of Sciences (SIMIT)

Measurement Report

Report No. 24TR060402

**Client Name**	Wuhan University of Technology, Jinhui Tong Group
**Client Address**	122 Luoshi Road, Hongshan District, Wuhan, Hubei, China
**Sample**	Perovskite/perovskite Tandem Solar Cell
**Manufacturer**	Wuhan University of Technology
**Measurement Date**	4th June, 2024

**Performed by:**	Qiang Shi *Qiang Shi*	**Date:** 04/06/2024
**Reviewed by:**	Wenjie Zhao *Wenjie Zhao*	**Date:** 04/06/2024
**Approved by:**	Zhengxin Liu *Zhengxin Liu*	**Date:** 04/06/2024.

Address: No.235 Chengbei Road, Jiading, Shanghai **Post Code:**201800
E-mail: solarcell@mail.sim.ac.cn **Tel:** +86-021-69976905

The measurement report without signature and seal are not valid.
This report shall not be reproduced, except in full, without the approval of SIMIT.

Sample Information

Sample Type	Perovskite/perovskite tandem solar cell
Serial No.	0531-8-3
Lab Internal No.	24060401-2#
Measurement Item	I-V characteristic
Measurement Environment	24.6±2.0°C, 42.3±5.0%R.H

Measurement of I-V characteristic

**Reference cell**	PVM1121
**Reference cell Type**	mono-Si, WPVS, calibrated by NREL (Certificate No. ISO 2098)
**Calibration Value/Date of Calibration for Reference cell**	143.95mA/ Feb. 2024
**Measurement Conditions**	Standard Test Condition (STC):<br>Spectral Distribution: AM1.5 according to IEC 60904-3 Ed.3,<br>Irradiance: 1000±50W/m², Temperature: 25±2°C
**Measurement Equipment/ Date of Calibration**	AAA Steady State Solar Simulator (YSS-T155-2M) / July.2023<br>IV test system (ADCMT 6246) / June. 2023<br>Measuring Microscope (MF-B2017C) / July.2023<br>SR Measurement system (CEP-25ML-CAS) / May.2024
**Measurement Method**	I-V Measurement:<br>Logarithmic sweep in both directions (Voc to Isc and Isc to Voc) during one flash based on IEC 60904-1:2020;<br>Spectral Mismatch factor was calculated according to IEC 60904-7 and I-V correction according to IEC 60891.
**Measurement Uncertainty**	Area: 1.0%(k=2); Isc: 2.1%(k=2); Voc: 1.0%(k=2);<br>Pmax: 2.9%(k=2); Eff: 3.0%(k=2)

海微系 检测专

SIMIT

Report No. 24TR060402

====Measurement Results ====

	Forward Scan (Isc to Voc)	Reverse Scan (Voc to Isc)
Area	9.09 mm^2	
Isc	1.453 mA	1.455 mA
Voc	2.164 V	2.172 V
Pmax	2.525 mW	2.592 mW
Ipm	1.350 mA	1.372 mA
Vpm	1.870 V	1.890 V
FF	80.31 %	82.01 %
Eff	27.78 %	28.52 %

- Spectral Mismatch Factor: SMM_{top}=0.9952, SMM_{bot}=0.9948.
- Designated illumination area defined by a thin mask was measured by measuring microscope.
- Test results listed in this measurement report refer exclusively to the mentioned measured sample.
- The results apply only at the time of the test, and do not imply future performance.

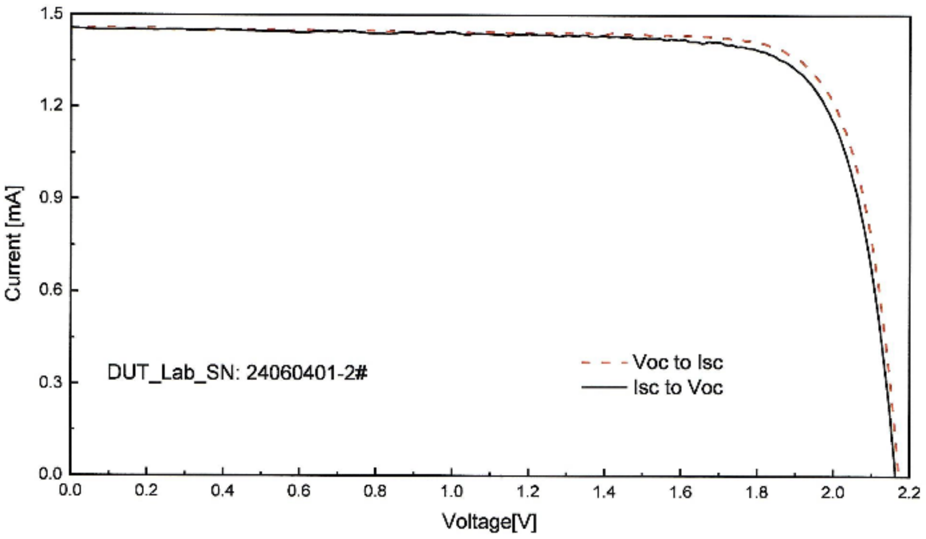

Fig.1 I-V curves of the measured sample

-------**End of Report**-------

Fig. S32. Certification report for a representative MA-free all-perovskite tandem solar cell based on a TFBZ-modified 1.79 eV WBG PSC subcell by Shanghai Institute of Microsystem and Information Technology (SIMIT).

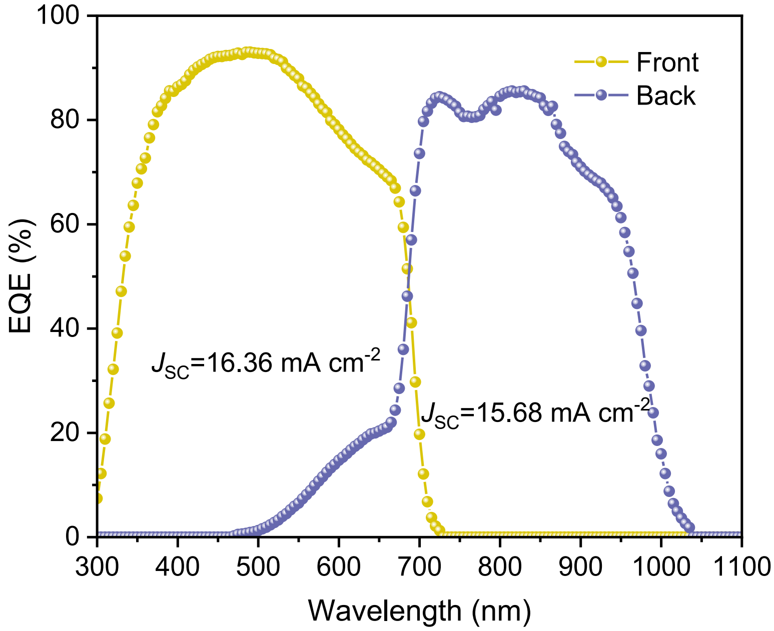

Fig. S33. EQE curves of the MA-free all-perovskite tandem solar cell.

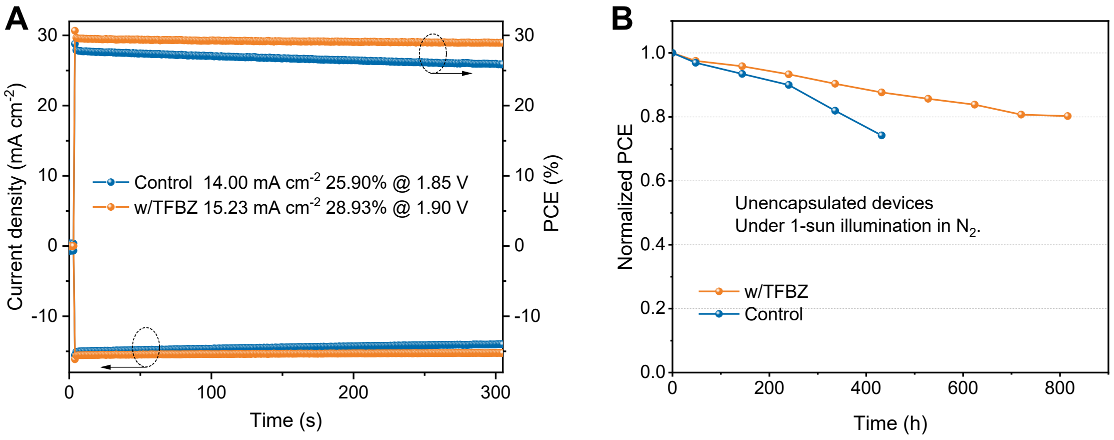

Fig. S34. Stability of the all-perovskite TSCs. (A) SPO curves of the control device and the w/TFBZ device. **(B)** Stability of unencapsulated all-perovskite tandem devices under N_2 conditions at simulated 1 sun illumination.

Table S1. Dipole moments of BZD and TFBZ.

Sample	Dipole moment (*D*)
BZD	3.616
TFBZ	4.475

Table S2. The FWHM of the (100) diffraction peak for the control, BZD-based and TFBZ-based perovskite films.

Sample	FWHM (°)
Control	0.154
w/BZD	0.136
w/TFBZ	0.107

Table S3. Fitting results of TR.

Sample	D*10^{-5} (m^{2}s^{-1})	S (ms^{-1})
Control	1.03	12.20
w/BZD	4.36	7.51
w/TFBZ	7.18	2.92

Table S4. The carrier mobilities and carrier diffusion lengths of the control, w/BZD, and w/TFBZ perovskite films.

Sample	$\varphi\Sigma\mu$ ($cm^2V^{-1}s^{-1}$)	L_D (μm)
Control	17.51 ± 0.4	6.96 ± 0.95
w/BZD	19.22 ± 0.8	7.12 ± 0.86
w/TFBZ	21.23 ± 0.5	7.70 ± 0.99

Note: $L_D=\left(\frac{\mu k_B T}{e k_1}\right)^{\frac{1}{2}}$, where k_B represents the Boltzmann constant, T denotes the temperature, μ is carrier mobility, and e signifies the elementary charge.

Table S5. The carrier lifetimes extracted from the TRPL spectra by the bi-exponential rate law of the control, w/BZD, and w/TFBZ perovskite films deposited on glass.

Sample	τ_1 (μs)	A_1 (%)	τ_2 (μs)	A_2 (%)	τ_{ave} (μs)
Control	0.21	0.33	1.03	0.20	0.83
w/BZD	0.15	0.66	1.58	0.17	1.21
w/TFBZ	0.47	0.21	1.86	0.21	1.58

Note: The PL lifetime τ_1 and τ_2 are extracted by fitting with a bi-exponential decay function of $I(t)=A_1e^{-\frac{t}{\tau_1}}+A_2e^{-\frac{t}{\tau_2}}$, The τ_{ave} is calculated by the formula of $\tau_{ave}=\frac{A_1\tau_1{}^2+A_2\tau_2{}^2}{A_1\tau_1+A_2\tau_2}$.

Table S6. PV parameters of 1.67 eV WBG PSCs with varying BZD concentrations.

BZD (mol%)	V_{OC} (V)	J_{SC} (mA cm^{-2})	FF (%)	PCE (%)
Control	1.20	19.78	80.50	19.08
w/BZD (0.05%)	1.24	20.57	81.26	20.67
**w/BZD (0.1%)**	**1.25**	**20.74**	**81.74**	**21.17**
w/BZD (0.15%)	1.26	20.86	79.60	20.94
w/BZD (0.2%)	1.26	20.67	77.84	20.37
w/BZD (0.25%)	1.26	20.88	77.09	20.25

Table S7. PV parameters of 1.67 eV WBG PSCs with varying TFBZ concentrations.

TFBZ (mol%)	V_{OC} (V)	J_{SC} (mA cm^{-2})	FF (%)	PCE (%)
Control	1.24	20.21	77.00	19.40
w/TFBZ (0.04%)	1.26	20.71	81.17	21.18
w/TFBZ (0.08%)	1.28	21.10	83.64	22.51
**w/TFBZ (0.12%)**	**1.28**	**21.13**	**83.71**	**22.62**
w/TFBZ (0.16%)	1.28	21.13	82.18	22.16
w/TFBZ (0.2%)	1.27	21.08	80.57	21.57

Table S8. The calculated V_{OC} loss analysis results of the 1.67 eV WBG PSCs.

Sample	E_g (eV)	V_{OC}^{SQ} (V)	V_{OC} (V)	ΔV_1 (mV)	ΔV_2 (mV)	ΔV_3 (mV)	V_{OC}* (V)
Control	1.669	1.391	1.253	278	22	116	1.253
w/TFBZ	1.669	1.392	1.279	277	22	86	1.284

Note: $q\Delta V=E_g-qV_{OC}=q(\Delta V_1+\Delta V_2+\Delta V_3)$, where q is the elementary charge, ΔV is the overall voltage loss, and E_g is the perovskite band gap. ΔV_1 is the radiative recombination voltage loss, ΔV_2 is the blackbody radiation voltage loss, and ΔV_3 is the non-radiative recombination voltage loss.

V_{OC}^{SQ} is the Shockley−Queisser limit of V_{OC}.

V_{OC} is the measured value extracted from the *J-V* curve.

V_{OC}* is the V_{OC} calculated by subtracting ΔV_1, ΔV_2, and ΔV_3 from E_g.

Table S9. Electrochemical impedance analysis data of the 1.67 eV WBG PSCs.

Device	R_s (ohm)	C_{rec} (nF)	R_{rec} (ohm)
Control	54.2	8.6	8414
w/BZD	28.1	8.1	9614
w/TFBZ	25.9	8.6	11797

Table S10. PV parameters of the 1.79 eV WBG PSCs. The devices were measured with an active area of 0.09 cm^2 under 1 sun illumination.

Device	Scan direction	V_{OC} (V)	J_{SC} (mA cm^{-2})	FF (%)	PCE (%)
Control	Reverse	1.30	17.35	80.06	18.07
	Forward	1.30	17.45	79.21	17.97
w/TFBZ	Reverse	1.36	17.97	82.48	20.21
	Forward	1.36	18.03	80.03	19.68

Table S11. Summary of reported high-efficient WBG PSCs with bandgaps between 1.75 and 1.80 eV.

E_g (eV)	V_{OC} (V)	J_{SC} (mA cm^{-2})	FF (%)	PCE (%)	Reference
1.75	1.25	16.9	83.00	17.6	Adv. Mater. 34, 2108829 (2022). (*45*)
1.75	1.29	18.0	84.8	19.7	Adv. Mater. 36, 2307701 (2024). (*46*)
1.77	1.25	18.79	83.7	19.66	Small 19, 2303213 (2023). (*47*)
1.77	1.26	18.17	83.53	19.28	Small 20, 2401197 (2024). (*48*)
1.77	1.29	18.01	83.08	19.30	Adv. Energy Mater. 14, 2304429 (2024). (*49*)
1.77	1.23	18.1	82.8	18.5	Nat. Energy 8, 610-620 (2023). (*2*)
1.77	1.25	17.2	80.00	17.4	Adv. Mater. 35, 2211742 (2023). (*50*)
1.77	1.33	16.43	80.94	17.72	Adv. Funct. Mater. 33, 2308794 (2023). (*51*)
1.77	1.33	17.75	82.7	19.53	Energy Environ. Sci. 17, 202-209 (2024). (*52*)
1.77	1.30	18.6	83.0	20.1	Nature 625, 516-522 (2024). (*53*)
1.77	1.35	17.2	80.9	18.7	Science 384,767-775 (2024). (*37*)
1.78	1.31	17.2	83.0	18.7	Adv. Mater. 36, 2307701 (2024). (*46*)
1.78	1.32	17.9	83.0	19.6	Nat. Commun. 14, 7118 (2023). (*54*)
1.78	1.35	17.65	83.11	19.83	Energy Environ. Sci. 16, 5992-6002 (2023). (*55*)
1.78	1.35	17.5	82.0	19.4	ACS Energy Lett. 9, 1984-1992 (2024). (*56*)
1.79	1.34	17.80	83.10	19.53	Adv. Mater. 36, 2306568 (2024). (*8*)
1.79	1.33	18.06	84.2	20.2	Nature 613, 676-681 (2023). (*57*)
**1.79**	**1.36**	**17.97**	**82.48**	**20.21**	**This work**

Table S12. Summary of reported high-efficient all-perovskite TSCs.

V_{OC} (V)	J_{SC} (mA cm^{-2})	FF (%)	PCE (%)	Reference
2.16	15.6	81	27.2	Nat. Commun. 15, 4136 (2024). (*58*)
2.08	16.48	81.4	27.6	Energy Environ. Sci. 17, 8557-8569 (2024). (*59*)
2.14	15.69	82.83	27.81	Sci. Adv. 10, eadl2063 (2024). (*60*)
2.13	16.06	84.19	28.80	Nat. Commun. 15, 7335 (2024). (*61*)
2.11	15.85	81.4	27.27	Nat. Energy 9, 298-307 (2024). (*62*)
2.12	15.84	81.01	27.20	Adv. Funct. Mater. 34, 2410605 (2024). (*63*)
2.10	15.68	82	27.03	Adv. Mater. 36, 2405860 (2024). (*64*)
2.15	15.5	80.2	26.8	Science, 383, 855-859 (2024). (*13*)
2.11	15.8	81	27	ACS Energy Lett. 9, 1984-1992 (2024). (*56*)
2.12	16.01	80.36	27.23	Nat. Sci. Rev. 11, nwae055 (2024). (*65*)
2.13	15.56	83.89	27.85	Adv. Mater. 36, 2401698 (2024). (*66*)
2.12	15.78	78.7	26.33	Energy Environ. Sci. 17, 2512-2520 (2024). (*67*)
**2.19**	**16.08**	**82.58**	**29.01**	**This work**

Table S13. Summary of reported high-efficient MA-free all-perovskite TSCs.

V_{OC} (V)	J_{SC} (mA cm^{-2})	FF (%)	PCE (%)	Reference
2.13	15.58	82.6	27.41	Adv. Energy Mater. 14, 2402171 (2024). (*68*)
2.06	15.2	76.9	24.1	ACS Energy Lett. 8, 2728-2737 (2023). (*69*)
2.04	15.1	82.1	25.3	Adv. Energy Mater. 13, 2300968 (2023). (*70*)
2.05	16.0	80.1	26.2	Adv. Mater. 34, 2110356 (2022). (*71*)
2.03	15.4	78.1	24.4	Sci. Adv. 8, eadd0377 (2022). (*72*)
1.77	15.83	74.17	20.8	Nat Energy 4, 939-947 (2019). (*73*)
1.88	16.0	77	23.1	Joule 3, 2193-2204 (2019). (*74*)
**2.19**	**16.08**	**82.58**	**29.01**	**This work**

REFERENCES AND NOTES

1. Z. Zhang, W. Chen, X. Jiang, J. Cao, H. Yang, H. Chen, F. Yang, Y. Shen, H. Yang, Q. Cheng, X. Chen, X. Tang, S. Kang, X.-m. Ou, C. J. Brabec, Y. Li, Y. Li, Suppression of phase segregation in wide-bandgap perovskites with thiocyanate ions for perovskite/organic tandems with 25.06% efficiency. *Nat. Energy* **9**, 592–601 (2024).

2. T. Li, J. Xu, R. Lin, S. Teale, H. Li, Z. Liu, C. Duan, Q. Zhao, K. Xiao, P. Wu, B. Chen, S. Jiang, S. Xiong, H. Luo, S. Wan, L. Li, Q. Bao, Y. Tian, X. Gao, J. Xie, E. H. Sargent, H. Tan, Inorganic wide-bandgap perovskite subcells with dipole bridge for all-perovskite tandems. *Nat. Energy* **8**, 610–620 (2023).

3. D. Kim, H. J. Jung, I. J. Park, B. W. Larson, S. P. Dunfield, C. Xiao, J. Kim, J. Tong, P. Boonmongkolras, S. G. Ji, F. Zhang, S. R. Pae, M. Kim, S. B. Kang, V. Dravid, J. J. Berry, J. Y. Kim, K. Zhu, D. H. Kim, B. Shin, Efficient, stable silicon tandem cells enabled by anion-engineered wide-bandgap perovskites. *Science* **368**, 155–160 (2020).

4. A. J. Ramadan, R. D. J. Oliver, M. B. Johnston, H. J. Snaith, Methylammonium-free wide-bandgap metal halide perovskites for tandem photovoltaics. *Nat. Rev. Mater.* **8**, 822–838 (2023).

5. Y. Zheng, C. Tian, X. Wu, A. Sun, R. Zhuang, C. Tang, Y. Liu, Z. Li, B. Ouyang, J. Du, Z. Li, X. Wu, J. Chen, J. Cai, C.-C. Chen, Dual-interface modification for inverted methylammonium-free perovskite solar cells of 25.35% efficiency with balanced crystallization. *Adv. Energy Mater.* **14**, 2304486 (2024).

6. S. Wang, L. Tan, J. Zhou, M. Li, X. Zhao, H. Li, W. Tress, L. Ding, M. Graetzel, C. Yi, Over 24% efficient MA-free $Cs_xFA_{1-x}PbX_3$ perovskite solar cells. *Joule* **6**, 1344–1356 (2022).

7. K. Wang, Z. Xu, Z. Guo, H. Wang, S. M. H. Qaid, K. Yang, Z. Zang, Phosphonate diacid molecule induced crystallization manipulation and defect passivation for high-performance inverted MA-free perovskite solar cells. *Adv. Energy Mater.* **14**, 2402249 (2024).

8. Y. An, N. Zhang, Z. Zeng, Y. Cai, W. Jiang, F. Qi, L. Ke, F. R. Lin, S.-W. Tsang, T. Shi, A. K. Y. Jen, H.-L. Yip, Optimizing crystallization in wide-bandgap mixed halide perovskites for high-efficiency solar cells. *Adv. Mater.* **36**, e2306568 (2024).

9. Q. Jiang, J. Tong, R. A. Scheidt, X. Wang, A. E. Louks, Y. Xian, R. Tirawat, A. F. Palmstrom, M. P. Hautzinger, S. P. Harvey, S. Johnston, L. T. Schelhas, B. W. Larson, E. L. Warren, M. C. Beard, J. J. Berry, Y. Yan, K. Zhu, Compositional texture engineering for highly stable wide-bandgap perovskite solar cells. *Science* **378**, 1295–1300 (2022).

10. R. Wang, X. Liu, S. Yan, N. Meng, X. Zhao, Y. Chen, H. Li, S. M. H. Qaid, S. Yang, M. Yuan, T. He, Efficient wide-bandgap perovskite photovoltaics with homogeneous halogen-phase distribution. *Nat. Commun.* **15**, 8899 (2024).

11. F. Li, X. Deng, Z. Shi, S. Wu, Z. Zeng, D. Wang, Y. Li, F. Qi, Z. Zhang, Z. Yang, S.-H. Jang, F. R. Lin, S. W. Tsang, X.-K. Chen, A. K. Y. Jen, Hydrogen-bond-bridged intermediate for perovskite solar cells with enhanced efficiency and stability. *Nat. Photonics* **17**, 478–484 (2023).

12. J. Tao, C. Zhao, Z. Wang, Y. Chen, L. Zang, G. Yang, Y. Bai, J. Chu, Suppressing non-radiative recombination for efficient and stable perovskite solar cells. *Energ. Environ. Sci.* **18**, 509–544 (2025).

13. H. Gao, K. Xiao, R. Lin, S. Zhao, W. Wang, S. Dayneko, C. Duan, C. Ji, H. Sun, A. D. Bui, C. Liu, J. Wen, W. Kong, H. Luo, X. Zheng, Z. Liu, H. Nguyen, J. Xie, L. Li, M. I. Saidaminov, H. Tan, Homogeneous crystallization and buried interface passivation for perovskite tandem solar modules. *Science* **383**, 855–859 (2024).

14. S. Nagane, S. Macpherson, M. A. Hope, D. J. Kubicki, W. Li, S. D. Verma, J. Ferrer Orri, Y.-H. Chiang, J. L. MacManus-Driscoll, C. P. Grey, S. D. Stranks, Tetrafluoroborate-induced reduction in defect density in hybrid perovskites through halide management. *Adv. Mater.* **33**, e2102462 (2021).

15. Y. Yao, B. Li, D. Ding, C. Kan, P. Hang, D. Zhang, Z. Hu, Z. Ni, X. Yu, D. Yang, Oriented wide-bandgap perovskites for monolithic silicon-based tandems with over 1000 hours operational stability. *Nat. Commun.* **16**, 40 (2025).

16. Y. Yu, C. Wang, C. R. Grice, N. Shrestha, D. Zhao, W. Liao, L. Guan, R. A. Awni, W. Meng, A. J. Cimaroli, K. Zhu, R. J. Ellingson, Y. Yan, Synergistic effects of lead thiocyanate additive and solvent annealing on the performance of wide-bandgap perovskite solar cells. *ACS Energy Lett.* **2**, 1177–1182 (2017).

17. P. Pandey, S. Cho, J. Bahadur, S. Yoon, C.-M. Oh, I.-W. Hwang, H. Song, H. Choi, S. Hayase, J. S. Cho, D.-W. Kang, 4-Phenylthiosemicarbazide molecular additive engineering for wide-bandgap Sn halide perovskite solar cells with a record efficiency over 12.2%. *Adv. Energy Mater.* **14**, 2401188 (2024).

18. X. Jiang, L. Zhu, B. Zhang, L. Zheng, L. Wang, P. Li, M. Wang, G. Yang, K. Dong, S. Li, S. Liu, Y. Yin, H. Wang, S. M. Zakeeruddin, S. Pang, L. Sun, M. Grätzel, X. Guo, Spatial conformation engineering of aromatic ketones for highly efficient and stable perovskite solar cells. *J. Am. Chem. Soc.* **146**, 34833–34841 (2024).

19. X. Guo, Z. Jia, S. Liu, R. Guo, F. Jiang, Y. Shi, Z. Dong, R. Luo, Y.-D. Wang, Z. Shi, J. Li, J. Chen, L. K. Lee, P. Müller-Buschbaum, D. S. Ginger, D. J. Paterson, Y. Hou, Stabilizing efficient wide-bandgap perovskite in perovskite-organic tandem solar cells. *Joule* **8**, 2554–2569 (2024).

20. R. Sun, Q. Tian, M. Li, H. Wang, J. Chang, W. Xu, Z. Li, Y. Pan, F. Wang, T. Qin, Over 24% efficient poly(vinylidene fluoride) (PVDF)-coordinated perovskite solar cells with a photovoltage up to 1.22 V. *Adv. Funct. Mater.* **33**, 2210071 (2023).

21. Y. Yu, R. Liu, C. Liu, X.-L. Shi, H. Yu, Z.-G. Chen, Synergetic regulation of oriented crystallization and interfacial passivation enables 19.1% efficient wide-bandgap perovskite solar cells. *Adv. Energy Mater.* **12**, 2201509 (2022).

22. Y. Wang, R. Lin, C. Liu, X. Wang, C. Chosy, Y. Haruta, A. D. Bui, M. Li, H. Sun, X. Zheng, H. Luo, P. Wu, H. Gao, W. Sun, Y. Nie, H. Zhu, K. Zhou, H. T. Nguyen, X. Luo, L. Li, C. Xiao, M. I. Saidaminov, S. D. Stranks, L. Zhang, H. Tan, Homogenized contact in all-perovskite tandems using tailored 2D perovskite. *Nature* **635**, 867–873 (2024).

23. P. Hu, W. Zhou, J. Chen, X. Xie, J. Zhu, Y. Zheng, Y. Li, J. Li, M. Wei, Multidentate anchoring strategy for synergistically modulating crystallization and stability towards efficient perovskite solar cells. *Chem. Eng. J.* **480**, 148249 (2024).

24. S. Wang, P. Wang, B. Shi, C. Sun, H. Sun, S. Qi, Q. Huang, S. Xu, Y. Zhao, X. Zhang, Inorganic perovskite surface reconfiguration for stable inverted solar cells with 20.38% efficiency and its application in tandem devices. *Adv. Mater.* **35**, e2300581 (2023).

25. R. Wang, J. Xue, K.-L. Wang, Z.-K. Wang, Y. Luo, D. Fenning, G. Xu, S. Nuryyeva, T. Huang, Y. Zhao, J. L. Yang, J. Zhu, M. Wang, S. Tan, I. Yavuz, K. N. Houk, Y. Yang, Constructive molecular configurations for surface-defect passivation of perovskite photovoltaics. *Science* **366**, 1509–1513 (2019).

26. Z. Li, J. Wang, S. Lu, J. Liu, J. Zeng, H. Gao, C. Liu, W. Guo, Targeted synergistic chemical bonding strategy for efficient and stable $CsPbI_3$-based perovskite solar cells. *Chem. Eng. J.* **499**, 156691 (2024).

27. S. Zhou, S. Fu, C. Wang, W. Meng, J. Zhou, Y. Zou, Q. Lin, L. Huang, W. Zhang, G. Zeng, D. Pu, H. Guan, C. Wang, K. Dong, H. Cui, S. Wang, T. Wang, G. Fang, W. Ke, Aspartate all-in-one doping strategy enables efficient all-perovskite tandems. *Nature* **624**, 69–73 (2023).

28. L. Lin, T. W. Jones, T. C.-J. Yang, X. Li, C. Wu, Z. Xiao, H. Li, J. Li, J. Qian, L. Lin, J. Q. Shi, S. D. Stranks, G. J. Wilson, X. Wang, Hydrogen bonding in perovskite solar cells. *Matter* **7**, 38–58 (2024).

29. Y. Kong, W. Shen, H. Cai, W. Dong, C. Bai, J. Zhao, F. Huang, Y. B. Cheng, J. Zhong, Multifunctional organic potassium salt additives as the efficient defect passivator for high-efficiency and stable perovskite solar cells. *Adv. Funct. Mater.* **33**, 2300932 (2023).

30. M. Li, R. Sun, J. Chang, J. Dong, Q. Tian, H. Wang, Z. Li, P. Yang, H. Shi, C. Yang, Z. Wu, R. Li, Y. Yang, A. Wang, S. Zhang, F. Wang, W. Huang, T. Qin, Orientated crystallization of FA-based perovskite via hydrogen-bonded polymer network for efficient and stable solar cells. *Nat. Commun.* **14**, 573 (2023).

31. W. Yue, H. Yang, H. Cai, Y. Xiong, T. Zhou, Y. Liu, J. Zhao, F. Huang, Y.-B. Cheng, J. Zhong, Printable high-efficiency and stable $FAPbBr_3$ perovskite solar cells for multifunctional building-integrated photovoltaics. *Adv. Mater.* **35**, e2301548 (2023).

32. T. Bu, J. Li, H. Li, C. Tian, J. Su, G. Tong, L. K. Ono, C. Wang, Z. Lin, N. Chai, X.-L. Zhang, J. Chang, J. Lu, J. Zhong, W. Huang, Y. Qi, Y.-B. Cheng, F. Huang, Lead halide–templated crystallization of methylamine-free perovskite for efficient photovoltaic modules. *Science* **372**, 1327–1332 (2021).

33. Y. Zhao, F. Ma, Z. Qu, S. Yu, T. Shen, H.-X. Deng, X. Chu, X. Peng, Y. Yuan, X. Zhang, J. You, Inactive $(PbI_2)_2RbCl$ stabilizes perovskite films for efficient solar cells. *Science* **377**, 531–534 (2022).

34. Y. Ding, S. Lu, J. Chang, E. Feng, H. Li, C. Long, Y. Yang, C. Yi, Z. Zheng, L. Ding, J. Yang, The molecular additive N-acetyl-L-phenylalanine delays the crystallization and suppresses the phase impurity for achieving triple-cation perovskite solar cells with efficiency over 25%. *Small* **21**, e2410601 (2025).

35. H. Xu, Z. Liang, J. Ye, Y. Zhang, Z. Wang, H. Zhang, C. Wan, G. Xu, J. Zeng, B. Xu, Z. Xiao, T. Kirchartz, X. Pan, Constructing robust heterointerfaces for carrier viaduct via interfacial molecular bridges enables efficient and stable inverted perovskite solar cells. *Energ. Environ. Sci.* **16**, 5792–5804 (2023).

36. Y. Yuan, G. Yan, C. Dreessen, T. Rudolph, M. Hülsbeck, B. Klingebiel, J. Ye, U. Rau, T. Kirchartz, Shallow defects and variable photoluminescence decay times up to 280 μs in triple-cation perovskites. *Nat. Mater.* **23**, 391–397 (2024).

37. Y.-H. Lin, Vikram, F. Yang, X.-L. Cao, A. Dasgupta, R. D. J. Oliver, A. M. Ulatowski, M. M. McCarthy, X. Shen, Q. Yuan, M. G. Christoforo, F. S. Y. Yeung, M. B. Johnston, N. K. Noel, L. M. Herz, M. S. Islam, H. J. Snaith, Bandgap-universal passivation enables stable perovskite solar cells with low photovoltage loss. *Science* **384**, 767–775 (2024).

38. X. Yang, Y.-H. Huang, X.-D. Wang, W.-G. Li, D.-B. Kuang, A-site diamine cation anchoring enables efficient charge transfer and suppressed ion migration in Bi-based hybrid perovskite single crystals. *Angew. Chem. Int. Ed. Engl.* **61**, e202204663 (2022).

39. G. Yang, Z. Ni, Z. J. Yu, B. W. Larson, Z. Yu, B. Chen, A. Alasfour, X. Xiao, J. M. Luther, Z. C. Holman, J. Huang, Defect engineering in wide-bandgap perovskites for efficient perovskite–silicon tandem solar cells. *Nat. Photonics* **16**, 588–594 (2022).

40. F. Li, X. Deng, F. Qi, Z. Li, D. Liu, D. Shen, M. Qin, S. Wu, F. Lin, S.-H. Jang, J. Zhang, X. Lu, D. Lei, C.-S. Lee, Z. Zhu, A. K. Y. Jen, Regulating surface termination for efficient inverted perovskite solar cells with greater than 23% efficiency. *J. Am. Chem. Soc.* **142**, 20134–20142 (2020).

41. T. Ma, H. Wang, Z. Wu, Y. Zhao, C. Chen, X. Yin, L. Hu, F. Yao, Q. Lin, S. Wang, D. Zhao, X. Li, C. Wang, Hole transport layer-free low-bandgap perovskite solar cells for efficient all-perovskite tandems. *Adv. Mater.* **36**, 2308240 (2024).

42. Z. Wu, Y. Zhao, C. Wang, T. Ma, C. Chen, Y. Liu, T. Jia, Y. Zhai, C. Chen, C. Zhang, G. Cao, Z. Yang, D. Zhao, X. Li, Enhancing photovoltaically preferred orientation in wide-bandgap perovskite for efficient all-perovskite tandem solar cells. *Adv. Mater.* **37**, e2412943 (2025).

43. C. Jiang, T. Qin, L. Tan, H. Li, J. Zhou, M. Li, Z.-M. Dang, L. Ding, Q. Xiong, C. Yi, Revealing the hole and electron transport dynamics in the working devices for efficient semitransparent perovskite solar cells. *Adv. Energy Mater.* **14**, 2304093 (2024).

44. Q. Jiang, J. Tong, Y. Xian, R. A. Kerner, S. P. Dunfield, C. Xiao, R. A. Scheidt, D. Kuciauskas, X. Wang, M. P. Hautzinger, R. Tirawat, M. C. Beard, D. P. Fenning, J. J. Berry, B. W. Larson,

Y. Yan, K. Zhu, Surface reaction for efficient and stable inverted perovskite solar cells. *Nature* **611**, 278–283 (2022).

45. S. Qin, C. Lu, Z. Jia, Y. Wang, S. Li, W. Lai, P. Shi, R. Wang, C. Zhu, J. Du, J. Zhang, L. Meng, Y. Li, Constructing monolithic perovskite/organic tandem solar cell with efficiency of 22.0% via reduced open-circuit voltage loss and broadened absorption spectra. *Adv. Mater.* **34**, e2108829 (2022).

46. S. Li, Z. Zheng, J. Ju, S. Cheng, F. Chen, Z. Xue, L. Ma, Z. Wang, A generic strategy to stabilize wide bandgap perovskites for efficient tandem solar cells. *Adv. Mater.* **36**, 2307701 (2024).

47. Z. Zhang, J. Wang, J. Liang, Y. Zheng, X. Wu, C. Tian, A. Sun, Y. Huang, Z. Zhou, Y. Yang, Y. Liu, C. Tang, Z. Chen, C.-C. Chen, Organizing uniform phase distribution in methylammonium-free 1.77 eV wide-bandgap inverted perovskite solar cells. *Small* **19**, e2303213 (2023).

48. A. Zhang, M. Li, C. Dong, W. Ye, X. Yang, A. Shaker, M. S. Salem, Z. Li, J. Yang, X. Li, L. Xu, H. Song, C. Chen, J. Tang, π–π stacking at the perovskite/C_{60} interface enables high-efficiency wide-bandgap perovskite solar cells. *Small* **20**, 2401197 (2024).

49. Y. Luo, J. Zhu, X. Yin, W. Jiao, Z. Gao, Y. Xu, C. Wang, Y. Wang, H. Lai, H. Huang, J. Luo, J. Wang, J. You, Z. Zhang, X. Hao, G. Zeng, S. Ren, Z. Li, F. Fu, M. Li, C. Xiao, C. Chen, D. Zhao, Enhanced efficiency and stability of wide-bandgap perovskite solar cells via molecular modification with piperazinium salt. *Adv. Energy Mater.* **14**, 2304429 (2024).

50. X. Shen, B. M. Gallant, P. Holzhey, J. A. Smith, K. A. Elmestekawy, Z. Yuan, P. V. G. M. Rathnayake, S. Bernardi, A. Dasgupta, E. Kasparavicius, T. Malinauskas, P. Caprioglio, O. Shargaieva, Y.-H. Lin, M. M. McCarthy, E. Unger, V. Getautis, A. Widmer-Cooper, L. M. Herz, H. J. Snaith, Chloride-based additive engineering for efficient and stable wide-bandgap perovskite solar cells. *Adv. Mater.* **35**, e2211742 (2023).

51. G. Xie, H. Li, X. Wang, J. Fang, D. Lin, D. Wang, S. Li, S. He, L. Qiu, Phase segregation and voltage loss mitigated highly efficient perovskite–organic tandem solar cells with a simple ambipolar SnO_X interconnecting layer. *Adv. Funct. Mater.* **33**, 2308794 (2023).

52. Z. Yi, W. Wang, R. He, J. Zhu, W. Jiao, Y. Luo, Y. Xu, Y. Wang, Z. Zeng, K. Wei, J. Zhang, S.-W. Tsang, C. Chen, W. Tang, D. Zhao, Achieving a high open-circuit voltage of 1.339 V in 1.77 eV wide-bandgap perovskite solar cells via self-assembled monolayers. *Energ. Environ. Sci.* **17**, 202–209 (2024).

53. P. Chen, Y. Xiao, J. Hu, S. Li, D. Luo, R. Su, P. Caprioglio, P. Kaienburg, X. Jia, N. Chen, J. Wu, Y. Sui, P. Tang, H. Yan, T. Huang, M. Yu, Q. Li, L. Zhao, C.-H. Hou, Y.-W. You, J.-J. Shyue, D. Wang, X. Li, Q. Zhao, Q. Gong, Z.-H. Lu, H. J. Snaith, R. Zhu, Multifunctional ytterbium oxide buffer for perovskite solar cells. *Nature* **625**, 516–522 (2024).

54. J. Wen, Y. Zhao, P. Wu, Y. Liu, X. Zheng, R. Lin, S. Wan, K. Li, H. Luo, Y. Tian, L. Li, H. Tan, Heterojunction formed via 3D-to-2D perovskite conversion for photostable wide-bandgap perovskite solar cells. *Nat. Commun.* **14**, 7118 (2023).

55. H. Cui, L. Huang, S. Zhou, C. Wang, X. Hu, H. Guan, S. Wang, W. Shao, D. Pu, K. Dong, J. Zhou, P. Jia, W. Wang, C. Tao, W. Ke, G. Fang, Lead halide coordination competition at buried interfaces for low V_{OC}-deficits in wide-bandgap perovskite solar cells. *Energ. Environ. Sci.* **16**, 5992–6002 (2023).

56. J. Zhou, T. Wen, J. Sun, Z. Shi, C. Zou, Z. Shen, Y. Li, Y. Wang, Y. Lin, S. Yang, F. Liu, Z. Yang, Phase-stable wide-bandgap perovskites with 2D/3D structure for all-perovskite tandem solar cells. *ACS Energy Lett.* **9**, 1984–1992 (2024).

57. H. Chen, A. Maxwell, C. Li, S. Teale, B. Chen, T. Zhu, E. Ugur, G. Harrison, L. Grater, J. Wang, Z. Wang, L. Zeng, S. M. Park, L. Chen, P. Serles, R. A. Awni, B. Subedi, X. Zheng, C. Xiao, N. J. Podraza, T. Filleter, C. Liu, Y. Yang, J. M. Luther, S. De Wolf, M. G. Kanatzidis, Y. Yan, E. H. Sargent, Regulating surface potential maximizes voltage in all-perovskite tandems. *Nature* **613**, 676–681 (2023).

58. S. Tan, C. Li, C. Peng, W. Yan, H. Bu, H. Jiang, F. Yue, L. Zhang, H. Gao, Z. Zhou, Sustainable thermal regulation improves stability and efficiency in all-perovskite tandem solar cells. *Nat. Commun.* **15**, 4136 (2024).

59. Y. Bai, R. Tian, K. Sun, C. Liu, X. Lang, M. Yang, Y. Meng, C. Xiao, Y. Wang, X. Lu, J. Wang, H. Pan, Z. Song, S. Zhou, Z. Ge, Decoupling light-and oxygen-induced degradation mechanisms of Sn–Pb perovskites in all perovskite tandem solar cells. *Energ. Environ. Sci.* **17**, 8557–8569 (2024).

60. J. Zhu, Y. Xu, Y. Luo, J. Luo, R. He, C. Wang, Y. Wang, K. Wei, Z. Yi, Z. Gao, J. Wang, J. You, Z. Zhang, H. Lai, S. Ren, X. Liu, C. Xiao, C. Chen, J. Zhang, F. Fu, D. Zhao, Custom-tailored hole transport layer using oxalic acid for high-quality tin-lead perovskites and efficient all-perovskite tandems. *Sci. Adv.* **10**, eadl2063 (2024).

61. Y. Pan, J. Wang, Z. Sun, J. Zhang, Z. Zhou, C. Shi, S. Liu, F. Ren, R. Chen, Y. Cai, H. Sun, B. Liu, Z. Zhang, Z. Zhao, Z. Cai, X. Qin, Z. Zhao, Y. Ji, N. Li, W. Huang, Z. Liu, W. Chen, Surface chemical polishing and passivation minimize non-radiative recombination for all-perovskite tandem solar cells. *Nat. Commun.* **15**, 7335 (2024).

62. D. Yu, M. Pan, G. Liu, X. Jiang, X. Wen, W. Li, S. Chen, W. Zhou, H. Wang, Y. Lu, M. Ma, Z. Zang, P. Cheng, Q. Ji, F. Zheng, Z. Ning, Electron-withdrawing organic ligand for high-efficiency all-perovskite tandem solar cells. *Nat. Energy* **9**, 298–307 (2024).

63. W. Shen, H. Fang, D. Pu, W. Zheng, X. Zhang, G. Li, L. Huang, S. Zhou, W. Chen, Y. Zhou, Z. Feng, J. Liang, J. Zhou, P. Qin, G. Fang, W. Ke, Optimizing blade-coated tin–lead perovskite solar cells and tandems with multi-carboxyl and amino group integration. *Adv. Funct. Mater.* **34**, 2410605 (2024).

64. G. Liu, G. Yang, W. Feng, H. Li, M. Yang, Y. Zhong, X. Jiang, W.-Q. Wu, Regulating surface metal abundance via lattice-matched coordination for versatile and environmentally-viable Sn-Pb alloying perovskite solar cells. *Adv. Mater.* **36**, 2405860 (2024).

65. X. Jiang, Q. Zhou, Y. Lu, H. Liang, W. Li, Q. Wei, M. Pan, X. Wen, X. Wang, W. Zhou, D. Yu, H. Wang, N. Yin, H. Chen, H. Li, T. Pan, M. Ma, G. Liu, W. Zhou, Z. Su, Q. Chen, F. Fan, F. Zheng, X. Gao, Q. Ji, Z. Ning, Surface heterojunction based on n-type low-dimensional perovskite film for highly efficient perovskite tandem solar cells. *Nat. Sci. Rev.* **11**, nwae055 (2024).

66. G. Li, C. Wang, S. Fu, W. Zheng, W. Shen, P. Jia, L. Huang, S. Zhou, J. Zhou, C. Wang, H. Guan, Y. Zhou, X. Zhang, D. Pu, H. Fang, Q. Lin, W. Ai, W. Chen, G. Zeng, T. Wang, P. Qin, G. Fang, W. Ke, Boosting all-perovskite tandem solar cells by revitalizing the buried tin-lead perovskite interface. *Adv. Mater.* **36**, e2401698 (2024).

67. Q. Sun, Z. Zhang, H. Yu, J. Huang, X. Li, L. Dai, Q. Wang, Y. Shen, M. Wang, Surface charge transfer doping of narrow-bandgap Sn–Pb perovskites for high-performance tandem solar cells. *Energ. Environ. Sci.* **17**, 2512–2520 (2024).

68. J. Wang, Y. Pan, Z. Zhou, Q. Zhou, S. Liu, J. Zhang, C. Shi, R. Chen, Z. Zhao, Z. Cai, X. Qin, Z. Zhao, Z. Yang, Z. Liu, W. Chen, Bimolecular crystallization modulation boosts the efficiency and stability of methylammonium-free tin–lead perovskite and all-perovskite tandem solar cells. *Adv. Energy Mater.* **14**, 2402171 (2024).

69. Y.-H. Chiang, K. Frohna, H. Salway, A. Abfalterer, L. Pan, B. Roose, M. Anaya, S. D. Stranks, Vacuum-deposited wide-bandgap perovskite for all-perovskite tandem solar cells. *ACS Energy Lett.* **8**, 2728–2737 (2023).

70. J. Zhou, H. Qiu, T. Wen, Z. He, C. Zou, Y. Shi, L. Zhu, C.-C. Chen, G. Liu, S. Yang, F. Liu, Z. Yang, Acidity control of interface for improving stability of all-perovskite tandem solar cells. *Adv. Energy Mater.* **13**, 2300968 (2023).

71. J. Wen, Y. Zhao, Z. Liu, H. Gao, R. Lin, S. Wan, C. Ji, K. Xiao, Y. Gao, Y. Tian, J. Xie, C. J. Brabec, H. Tan, Steric engineering enables efficient and photostable wide-bandgap perovskites for all-perovskite tandem solar cells. *Adv. Mater.* **34**, e2110356 (2022).

72. B. Chen, Z. Yu, A. Onno, Z. Yu, S. Chen, J. Wang, Z. C. Holman, J. Huang, Bifacial all-perovskite tandem solar cells. *Sci. Adv.* **8**, eadd0377 (2022).

73. R. Prasanna, T. Leijtens, S. P. Dunfield, J. A. Raiford, E. J. Wolf, S. A. Swifter, J. Werner, G. E. Eperon, C. de Paula, A. F. Palmstrom, C. C. Boyd, M. F. A. M. van Hest, S. F. Bent, G. Teeter, J. J. Berry, M. D. McGehee, Design of low bandgap tin–lead halide perovskite solar cells to achieve thermal, atmospheric and operational stability. *Nat. Energy* **4**, 939–947 (2019).

74. A. F. Palmstrom, G. E. Eperon, T. Leijtens, R. Prasanna, S. N. Habisreutinger, W. Nemeth, E. A. Gaulding, S. P. Dunfield, M. Reese, S. Nanayakkara, T. Moot, J. Werner, J. Liu, B. To, S. T. Christensen, M. D. McGehee, M. F. A. M. van Hest, J. M. Luther, J. J. Berry, D. T. Moore, Enabling flexible all-perovskite tandem solar cells. *Joule* **3**, 2193–2204 (2019).
